# CREB-Regulated Transcriptional Coactivator 2 Proteome Landscape is Modulated by SREBF1

**DOI:** 10.1016/j.mcpro.2023.100637

**Published:** 2023-08-28

**Authors:** Jae Min Lim, Muhammad Ayaz Anwar, Hye-Sook Han, Seung-Hoi Koo, Kwang Pyo Kim

**Affiliations:** 1 Department of Applied Chemistry, Institute of Natural Science, Global Center for Pharmaceutical Ingredient Materials, Kyung Hee University, Yongin, South Korea; 2 Division of Life Sciences, Korea University, Seongbuk-Gu, Seoul, South Korea; 3 Department of Biomedical Science and Technology, Kyung Hee Medical Science Research Institute, Kyung Hee University, Seoul, South Korea

**Keywords:** coregulation, metabolism, mass spectrometry, PPI, STRING

## Abstract

cAMP response element-binding protein (CREB) regulated transcriptional coactivator 2 (CRTC2) is a critical transcription factor that maintains glucose homeostasis by activating CREB. Energy homeostasis is maintained through multiple pathways; therefore, CRTC2 may interact with other transcription factors, particularly under metabolic stress. CRTC2 liver-specific KO mice were created, and the global proteome, phosphoproteome, and acetylome from liver tissue under high-fat diet conditions were analyzed using liquid chromatography-tandem mass spectrometry and bioinformatics analysis. Differentially regulated proteins (DRPs) were enriched in metabolic pathways, which were subsequently corroborated through animal experiments. The consensus DRPs from these datasets were used as seed proteins to generate a protein-protein interaction network using STRING, and GeneMANIA identified fatty acid synthase as a mutually relevant protein. In an additional local-protein-protein interaction analysis of CRTC2 and fatty acid synthase with DRPs, sterol regulatory element binding transcription factor 1 (SREBF1) was the common mediator. CRTC2-CREB and SREBF1 are transcription factors, and DNA-binding motif analysis showed that multiple CRTC2-CREB–regulated genes possess SREBF1-binding motifs. This indicates the possible induction by the CRTC2–SREBF1 complex, which is validated through luciferase assay. Therefore, the CRTC2–SREBF1 complex potentially modulates the transcription of multiple proteins that fine-tune cellular metabolism under metabolic stress.

## Highlights

• Global proteome analysis of CRTC2^LKO^ revealed changes in metabolic-related pathways.
• Animal model also replicated altered glucose, fat deposits, and TG levels.
• Computational analysis showed that the altered metabolism is influenced by SREBF1.
• Knockout and luciferase validated the cooperation of CRTC2 and SREBF1 in metabolism.

## Graphical Abstract

**Figure:**
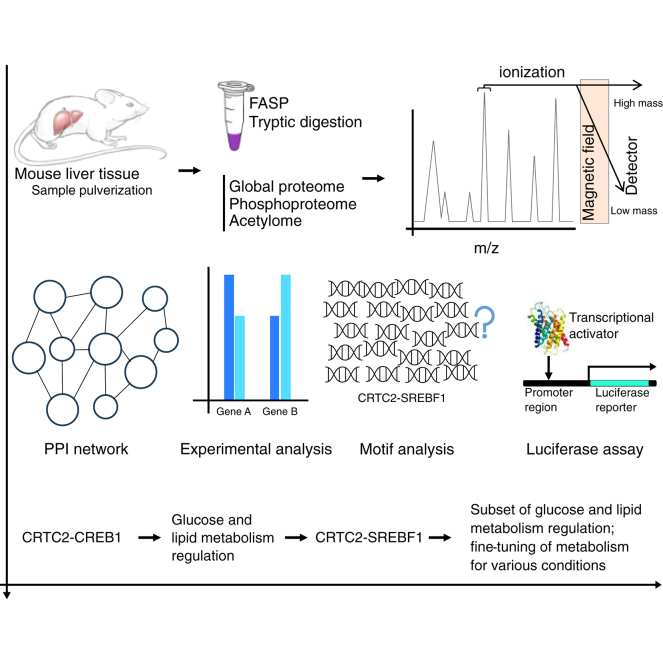

## In Brief

CRTC2 (with CREB1) in glucose metabolism is well studied; however, further studies are required to delineate the metabolic regulatory aspects. Here, using various biochemical and computational tools, it has been identified that CRTC2 along with SREBF1 regulates targeted genes to fine-tune the metabolic response under various conditions. This should further highlight the tight regulation of glucose metabolism and could be therapeutically used for various diseases.

Metabolic disorders, such as insulin resistance and type 2 diabetes, are on the rise and pose a risk of life-threatening challenges. Among the various factors, genetic predisposition, overnutrition, and sedentary lifestyle worsen the current disease landscape (1). In addition, the metabolic pathways, particularly hepatic lipogenesis, and gluconeogenesis, are consistently perturbed, contributing to insulin resistance and type 2 diabetes and further exacerbating the pathological condition (2, 3). These disorders are linked to perturbed metabolism, which is regulated by various signaling pathways and transcription factors. The pathways that regulate lipid metabolism include the liver X receptor, peroxisome proliferator-activated receptor gamma (PPARγ), carbohydrate response element binding protein, and sterol regulatory element-binding transcription factor 1 (SREBF1), which are regulated by cAMP response element-binding protein (CREB), forkhead box protein O1, PPARγ coactivator-1α, and CREB regulated transcription coactivators (CRTCs) at the transcriptional level (4, 5, 6, 7). CRTCs are extensively studied in glucose homeostasis (8, 9, 10); specifically, CRTC2 participates in glucose and lipid metabolism (11, 12). This makes CRTC2 pivotal in controlling energy metabolism; however, the detailed functional diversity needs to be elucidated. The progression of these disorders and highlighting the roles of various pathways and transcription factors have gained attention; however, further studies are required to delineate the underlying mechanism.

CRTC2 functions mainly as a transcriptional coactivator of CREB and controls hepatic glucose metabolism (11). Recently, it has been shown that CRTC2 is involved in SREBF1 processing, where it modulates COPII-dependent SREBF1 maturation. In addition, it regulates Insig2a in controlling SREBFP1 activity depending on the nutritional cues in mice (12, 13). CRTC2 induces microRNA 34a that negatively regulates the SIRT1–PPARα–FGF21 axis to regulate metabolic homeostasis (14). CRTC2 has a pivotal role in glucose and lipid metabolism; therefore, it is an attractive target for therapeutics against insulin resistance and diabetes (8).

The functional diversity of CRTC2 in energy homeostasis prompted the use of global proteomic data to underline the combination of key factors involved in energy metabolism. In this study, liver-specific *Crtc2* KO (Crtc2^LKO^) mice were generated and fed a high-fat diet (HFD), and global proteome, phosphoproteome, and acetylome changes in the liver were studied using LC-MS/MS analysis and protein-protein interaction (PPI) network. The diverse effects of Crtc2^LKO^ should involve other factors that in different stoichiometric combinations drive the expression of metabolic-related proteins as the derivative of this process is directly linked to a plethora of cellular functions.

## Experimental Procedures

### Experimental Design and Statistical Rationale

Three biological replicates of Crtc2^LKO^ and Crtc2^f/f^ tissues were used, and each pair of replicates (Crtc2^LKO^ and Crtc2^f/f^) were labeled with tandem mass tag (TMT) and pooled. From the pooled samples, 5% of labeled samples were used for global proteome analysis, and 95% were used for enrichment of phosphopeptides and acetylated peptides. Samples for global proteome analysis were divided into 12 fractions by high pH fractionation, while phosphopeptides and acetylated enriched peptide samples were divided into 12 and 6 fractions, respectively, using mid pH fractionation. All the fractions were subsequently injected into the LC-MS/MS system, and the raw mass spectra were processed by Proteome Discoverer v2.1 (Proteome Discoverer Software | Thermo Fisher Scientific - KR). The statistical significance of differential peptide expression has been measured using Student’s *t* test.

### Protein Extraction from Crtc2^LKO^ Mouse Tissue

*Crtc2* liver-specific KO (Crtc2^LKO^) mice were described previously (14). All animal experiments were performed in a specific pathogen-free facility at the Central Laboratory Animal Research Center, Korea University (12:12 h light-dark cycle, maintained at 22 °C), according to protocols approved by the Korea University Institutional Animal Care and Use Committee (KUIACUC-2018-0031). Mouse tissues given by Korea University were washed in PBS on ice to remove blood and were pulverized using a cryoPREP device (CP02, Covaris). Each tissue was placed in a cryovial (430487, Covaris) on liquid nitrogen, transferred to a Covaris tissue bag (TT1, Covaris), and pulverized at impact level 3 after placing the tissue bag into liquid nitrogen for 10 s. The pulverized tissue was subsequently placed in a sonication tube (002109, Covaris) and mixed with lysis buffer [4% SDS, 0.1 M Tris–HCl pH 7.6, and one tablet phosphatase inhibitor (04906845001, Sigma Aldrich) in 10 ml]. The tissue was lysed by sonication using a focused-ultrasonicator (S220, Covaris) at 2 W for 5 s, 36 W for 20 s, and 0 W for 10 s; the cycle was repeated 20 times at 16 °C. The lysate was centrifuged at 16,000*g* at 20 °C for 10 min; the supernatant was transferred to a new tube. To measure the protein concentration, a bicinchoninic acid protein assay (23227, Thermo Fisher Scientific) was used.

### Protein Digestion

Proteins (500 μg) from each tissue type were digested using the filter-aided sample preparation method. Proteins were reduced using SDT buffer (4% SDS in 0.1 M Tris–HCl, pH 7.6, and 0.1 M DTT) at 37 °C for 45 min and boiled for 15 min. The protein samples were sonicated for 15 min in a bath sonicator. The supernatant was transferred to a membrane filter (YM-30, Millipore) and centrifuged at 14,000*g* at 20 °C for 90 min to remove SDS. This procedure was performed thrice to completely remove SDS; subsequently, 100 μl of iodoacetamide buffer (0.05 M iodoacetamide in 8 M urea) was added to the filter and incubated for 30 min at room temperature in the dark, followed by centrifugation at 14,000*g* at 20 °C for 30 min. The buffer on the membrane filters was diluted with 200 μl of 8 M urea and concentrated thrice. Finally, 100 μl of 100 mM triethylammonium bicarbonate (pH 8.0) was added to the filter and centrifuged thrice at 14,000*g* at 20 °C for 30 min. Trypsin (90058, Thermo Fisher Scientific) was added at an enzyme to protein ratio of 1:50 (w/w), and the proteins were incubated at 37 °C for 12 h. The digested peptides were eluted through centrifugation at 14,000*g* and 20 °C for 30 min. To collect the residual peptides on the filter, the filter was washed thrice with 70 μl of 100 mM triethylammonium bicarbonate and centrifuged at 14,000*g* and 20 °C for 10 min. The eluents were dried and stored at −80 °C.

### TMT Labeling and High-pH Fractionation

The digested peptides were labeled with TMT isobaric tags (90061, Thermo Fisher Scientific) according to the manufacturer’s protocol. The two peptide samples from filter-aided sample preparation digestion were labeled with 126 and 129 for TMT reagents, where 126 for Crtc2^f/f^ and 129 for Crtc2^LKO^. All TMT-labeled peptides were pooled and concentrated using vacuum centrifugation. The labeled sample was concentrated to 200 μl and subjected to high-pH reverse-phase liquid chromatography fractionation. The TMT-labeled peptides were fractionated and concatenated into 12 fractions. Peptide samples were loaded on an analytical column (Xbridge, C18 5 μm, 4.6 mm × 250 mm) for separation with the linear gradient generated using an Agilent 1260 Infinity HPLC system. The mobile phase was composed of solvent A (10 mM ammonium hydroxide in water, pH 10.0) and solvent B (10 mM ammonium hydroxide in 90% ACN, pH 10.0). TMT-labeled peptides (2 mg) were diluted in 100 μl of solvent A, and the entire peptide solution was injected. A 115 min gradient with a flow rate of 0.5 ml/min was applied as follows: 100% solvent A for 10 min, 0% to 5% solvent B in 10 min, 5% to 35% over 60 min, 35% to 70% in 15 min, 70% for 10 min, and 70% to 0% in 10 min. The eluent was collected every 1 min between 15 and 110 min into 48 tubes at 4 °C. The 48 tube fractions were concatenated into 12 fractions by combining the four fractions from each section (*i.e.*, #1–#13–#25–#37, #2–#14–#26–#38, ... #12–#24–#36–#48). The concatenated 12 fractions were vacuum-dried and stored at −80 °C until LC-MS/MS analysis.

### Immobilized Metal Affinity Chromatography

Immobilized metal affinity chromatography beads were prepared from Ni-NTA magnetic beads (36113; Qiagen); 1.5 ml of Ni-NTA bead slurry was washed thrice with 1.2 ml of HPLC-grade water. To remove Ni2^+^, the beads were treated with 1.2 ml of 100 mM EDTA (pH 8.0) for 30 min using an end-over-end rotator (SB3; Stuart, Stone). After removing EDTA, the beads were rinsed thrice with 1.2 ml of water and treated with 1.2 ml of ice-cold 10 mM FeCl_3_ solution for 30 min with constant rotation. The resulting Fe^3+^-NTA beads were rinsed thrice with 1.2 ml of water, resuspended in 1.2 ml of 1:1:1 ACN/MeOH/0.01% acetic acid, and divided into 12 microcentrifuge tubes containing 100 μl bead solution each. Fe^3+^-NTA beads were washed with 400 μl binding buffer (80% ACN/0.1% TFA). TMT-labeled peptide samples of the 12 fractions were resuspended in 500 μl of binding buffer and transferred to a tube containing aliquoted beads. The binding reaction was allowed to proceed for 30 min with rotation; the reacted beads were rinsed with 500 μl binding buffer thrice; the phosphopeptides were eluted from the beads through incubation in 125 μl of 1:1 ACN/2.5% ammonia in 2 mM phosphate buffer (pH 10) for 1.5 min. The eluted phosphopeptides were acidified immediately with 10% TFA to pH 2.5 − 3.0 before vacuum drying.

### Enrichment of Acetylated Peptides through Immunoaffinity

For the analysis of acetylated peptides, flow-through peptides from the phospho-enrichment experiment were loaded into acetyl lysine antibody–enriched agarose beads (Cat. ICP0338, ImmuneChem). The antibody with captured peptides (130 μl) was diluted to 1.2 ml and centrifuged at 2000*g* for 10 min at 4 °C; the supernatant was removed, and this step was repeated thrice. The peptides (700 μl) were diluted to 1.3 ml with MOPS buffer, added to the antibody, and incubated for 12 h in a rotator. After centrifugation at 2000*g* for 10 min at 4 °C, the supernatant was collected. The peptides were washed thrice with 1 ml of MOPS buffer. Next, 100 μl of 0.15% TFA was added to elute the acetylated peptides. After incubation for 5 min, peptides were centrifuged at 2, 500*g* for 10 min at 4 °C. Eluted peptides were dried and resuspended in 0.1% formic acid for LC-MS/MS analysis.

To avoid any acetylation to carbamylation site modification, the experiments have been performed with caution such as (1) freshly prepared urea was used, and urea reagent was added to the mixture just before the experiment to avoid longer exposure and carbamylation, (2) urea was used only in alkylation step and immediately removed before the tryptic digestion, (3) heating the samples were avoided to prevent carbamylation, and finally, triethylammonium bicarbonate buffer (pH 7.6) was used before adding urea, which is a known buffer to prevent carbamylation reaction as reported previously (15).

### LC-MS/MS Analysis

The prepared samples (global, phospho-, and acetyl-profiling groups) were resuspended in 0.1% formic acid in water and analyzed using a Q Exactive Orbitrap hybrid mass spectrometer (Thermo Fisher Scientific) and an Easy-nLC1000 system (Thermo Fisher Scientific). The linear gradient was set up as follows: 5 to 40% of solvent B in 100 min, 40 to 80% of solvent B in 5 min, 80% of solvent B for 20 min, 80 to 5% of solvent B in 5 min, and equilibrating the column with 5% solvent B for 20 min. A trap column (2 cm × 75 μm packed with 2 μm C18 resin) and an analytical column (50 cm × 75 μm packed with 2 μm C18 resin) were used to separate the hydrophobic peptides. The data-dependent acquisition method was adopted, and the top 10 precursor peaks were selected and isolated for fragmentation. Ions were scanned at resolution of 70,000 in MS1 and 17,500 in MS2. The mass scan range was 400 to 2000 m/z at both MS1 and MS2 levels. The precursor ions were fragmented with an NCE of 30, and dynamic exclusion was set to 30 s.

### Raw Data Processing and Analysis

The raw data were preprocessed with post-experiment monoisotopic mass refinement to increase peptide identification sensitivity and accuracy by selecting a unique mass class. The refined data were analyzed using Proteome Discoverer version 2.1 (Thermo Fisher Scientific), where the mass spectral files were queried using the Sequest HT search engine against the UniProt mouse reference dataset (released in Feb 2016; total number of sequences were 16,680 without any contamination) with two missed cleavages that allowed using trypsin. While searching the database, carbamidomethylation, alkylation of S-bonds in cysteine (+57.021 Da), TMT 6-plex modification of lysine, and N-terminal of the peptide (+229.163 Da) were added as static modifications; oxidation of methionine (+15.995 Da) and phosphorylation of serine, threonine, and tyrosine (+79.966 Da) were added as variable modifications. The mass tolerance of precursor ions was chosen as 10 ppm, and the mass tolerance of MS/MS was set as 0.02 Da. The false discovery rate at the peptide level was kept at 1% for removing false positive peptide identification. To quantify each sample’s reporter ion, a “reporter ion quantifier” with a TMT 6-plex was used. Abundances of the TMT reporter ions were used at default parameters to quantify Crtc2^LKO^ and Crtc2^f/f^ group. For highly confident quantifications of proteins, the protein ratios were calculated from two or more unique quantitative peptides in each replicate. The detailed list of identified peptides and criteria for inclusion from global proteome, phosphoproteome, and acetylome have been provided in Supplemental Tables S1–S3, respectively.

### Identification of DAPs and Enrichment Analysis

The average value of triplicate protein intensity was used to calculate the log_2_ fold change (log2FC) as follows:

log2FC = log_2_ (protein intensity in Crtc2^LKO^/protein intensity in WT)

Proteins from proteome, phosphoproteome, and acetylome with log2FC > ±0.26 and a student t.test *p*-value of < 0.05 were considered as differentially abundant proteins (DAPs), differentially phosphorylated peptides (DPPs), and differentially acetylated peptides (DAcPs), respectively, and were used for gene ontology (GO) analysis and PPI generation. For GO enrichment analysis, either the DAPs from the independent dataset or all the differentially regulated proteins (DRPs) (a merge of DAPs from the independent dataset) were merged and used as input in EnrichR (16).

### PPI Network Using STRING

For the PPI, the Search Tool for the Retrieval of Interacting Genes/Proteins database (STRING v11.0) (17) was used, as provided in Cytoscape (v3.8.1) (18). The differentially abundant proteins from all three datasets (log2FC >±0.26, *p*-value < 0.05) were inputted, and the interactions were retrieved based on a confidence level of 0.4 with no additional interactors. STRING can create a PPI based on a variety of interaction types, such as physical interactions, localization, occurrences, and text mining, which are above the threshold.

### PPI Network Using GeneMANIA

The GeneMANIA tool (19, 20) can identify novel proteins from a given gene list for hypothesis testing. In addition, it extracts the PPI mode from various genomic and proteomic data. It calculates a score for the reliability of the possible interactions that can be used to prioritize the output gene list. While creating PPI using GeneMANIA, a mouse database was used with default interaction networks, and the top 25 partners and their attributes were extracted using different weighting methods for different PPIs.

### Functional Class Scoring Analysis

For functional class scoring, ClusterProfiler (v3.14.3) in R (v3.6.2) was used (21). A list of DAPs from the independent dataset with decreasing log2FC was provided, with a minimum gene set of 20 genes; the number of permutations was 5000; and the *p*-value was corrected using the Benjamini–Hochberg method. There were cases in which none of the pathways crossed the default *p*-value threshold (0.05); therefore, a lower value (cutoff value of 0.1) was used to analyze the top hit pathways.

### Signaling Pathway Impact Analysis

Signaling pathway impact analysis (SPIA) was performed to analyze pathway activation/inhibition based on upregulated or downregulated proteins (22). Only proteins common to the proteome, phosphoproteome, and acetylome were used. This analysis was performed in R (v3.6.2) with a mouse database, and the number of bootstrap iterations was set to 3000 for accurate results.

### Motif Analysis

Identification of motifs was performed in Rv3.6.2, using BioStrings (v2.58.0). The position frequency matrix for the CRE and E-box was retrieved from the mouse database, while the SRE1 human database was used, and the JASPAR2018 model (23) was used for all cases. Promoter sequences were extracted from the TxDB.Mmusculus.UCSC.mm10 annotation dataset based on genes; a sequence spanning ±5000 bases from the transcription start site was extracted using the GenomicFeatures library (24). The position weight matrix was compared against this sequence with 90% or above the match threshold.

### Branched-Chain Amino Acid Analysis

Crtc2^LKO^ and Crtc2^f/f^ tissues were obtained from Korea University. The weight of each tissue sample was measured; the tissues were pulverized with 500 μl of amino acid extraction buffer (acetonitrile: methanol: water, 40:40:20 v/v) using a centrifuge tube sample pestle. Standard branched-chain amino acid (BCAA) with heavy isotope labeling was spiked into each sample and incubated at −20 °C for 1 h. After incubation, samples were centrifuged at 16,000*g* for 10 min at 4 °C. The supernatants were collected, dried, and subjected to LC-MS/MS analysis using an Agilent Triple Quadrupole 6490. An XBridge BEH amide column was used for chromatographic separation with Sol A and Sol B (20 mM ammonium acetate, 20 mM ammonium hydroxide in 95:5 water: acetonitrile buffer at pH 9.45; 20 mM ammonium acetate, 20 mM ammonium hydroxide in acetonitrile at pH 9.45, respectively). A total of 30 min gradient with a flow rate of 0.25 ml/min was applied as follows: 85% of Sol B for 2 min, 15% to 20% of Sol A in 3 min, 20% to 25% of Sol A in 7 min, 25% to 30% of Sol A in 8 min, 30% to 50% of Sol A in 10 min, 50% to 75% of Sol A in 13 min, 75% to 100% of Sol A in 18 min, and 100% to 15% of Sol A in 24 min. The BCAAs transition was applied as follows: leucine light, 132.1/86; leucine heavy, 138.1/91.1; isoleucine, 132.1/86.1; isoleucine, 138.1/91; valine light, 118.1/76; and valine heavy, 123.1/72.

### Animal Experiments

For the liver disease mouse model maintained according to protocols approved by the Korea University Institutional Animal Care and Use Committee (KUIACUC-2018-0031), male mice were fed a 60% HFD (D12492, Research Diets) from 9 weeks of age for 8 weeks before being sacrificed under 4-h fasting conditions (from 9 AM to 1 PM) to eliminate any potential variations that are associated with differential feeding. The mice were euthanized by cervical dislocation to avoid any chemical contamination. For the glucose tolerance test, 16 h-fasted mice were intraperitoneally injected with a bolus of glucose (2 g/kg body weight). Blood glucose was measured in the tail vein blood using an automatic glucose monitor (One Touch, LifeScan). The livers were isolated from the mice and fixed using 10% formalin. Histological changes were examined by H&E staining. For Western blot analysis, antibodies against CRTC2 (ST1099, Sigma-Aldrich) and HSP90 (sc-7947; Santa Cruz Biotechnology) were used.

### Hepatic TG Quantification

Liver tissues were homogenized in 5% NP 40 solution. The concentration of triglyceride (TG) was measured using triglyceride quantification kit (Sigma) according to the manufacturer’s protocol. TG contents were normalized by the protein concentration (BCA protein assay kit, Bio-Rad).

### Luciferase Assays

293T cells were seeded on 24 well-plate, and then each expression plasmid (RSV-b-gal 50 ng, Fasn-pk-luciferase (−150/−43) 100 ng, pcDNA3-Srebp1c (1–436) 10 ng, pcDNA3-Crtc2 50 ng) was transfected into cells using TransIT-LT1 transfection reagent (Mirus) according to the manufacturer’s protocol. After 48 h of transfection, cells were treated with Forskolin 10 μM to activate CRTC2 for an additional 4 h before being harvested. The luciferase activity was measured by Luciferase Assay System (Promega) and normalized to the amount of β-galactosidase activity.

### siRNA Knockdown

AML12 hepatocyte cells were plated in 6-well plates. After 4 h of seeding, siRNA/liposome mixes containing 25 nM siRNA targeting mouse SREBP1 (Dharmacon) and 5 μl of Lipofectamine RNAiMAX (Invitrogen) were transfected for 72 h. Cells were harvested and mRNA and protein levels were analyzed.

### Primary Hepatocytes

Primary hepatocytes were prepared from either Crtc2^f/f^ mice or Crtc2^LKO^ mice as described previously and were cultured for 72 h before being harvested for the measurement of mRNA and protein levels.

### Statistical Analysis

Student’s *t* test was used to determine the significance between the differences in Crtc2^LKO^ and WT; a *p*-value < 0.05 was considered significant.

## Results

### Quantitative Analysis of Proteome, Phosphoproteome, and Acetylome of Liver Tissue from Crtc2 Liver-Specific KO (Crtc2^LKO^) Mice

**Fig. 1:**
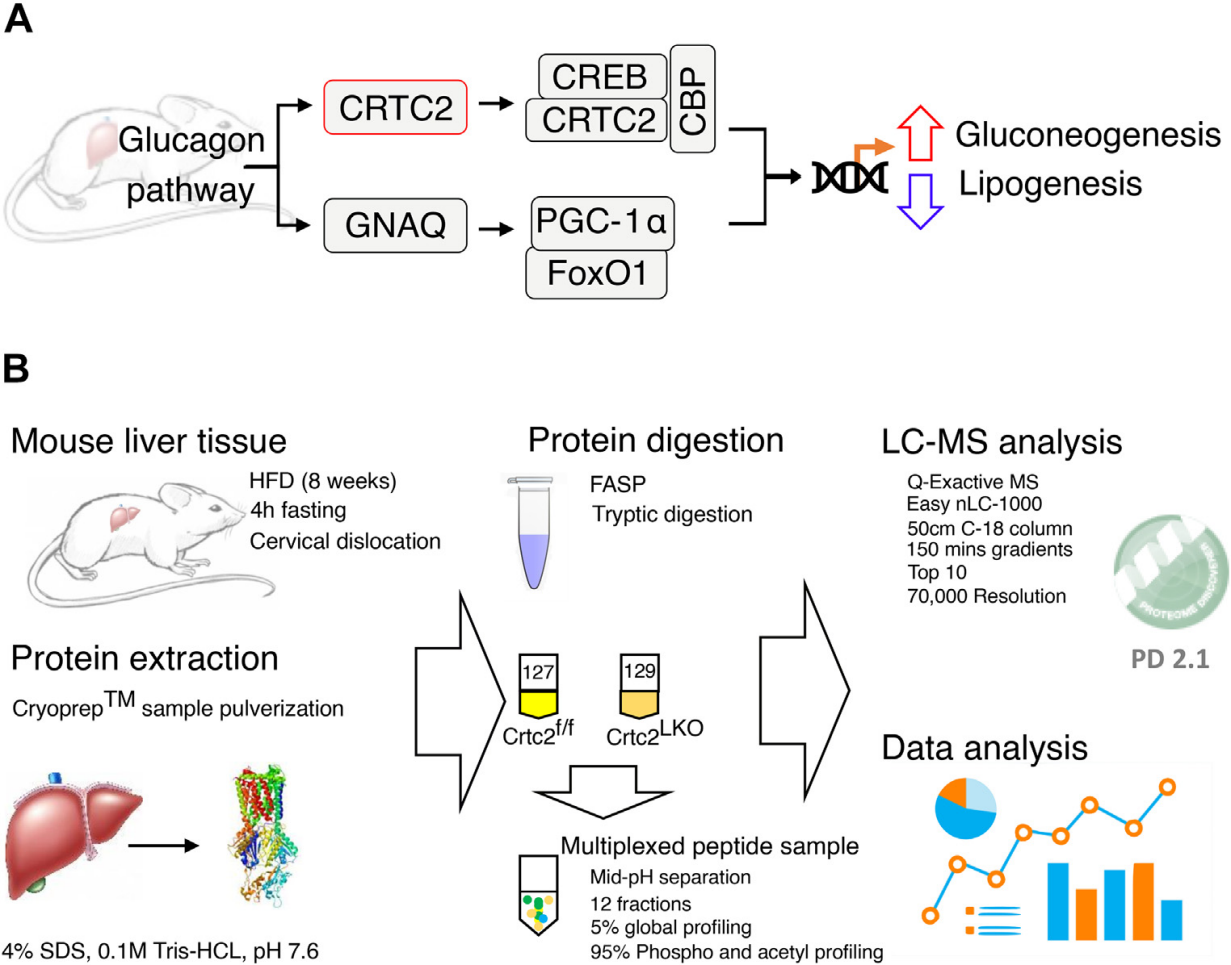
**Schema of the study.***A*, the glucagon pathway initiates when glucagon (GCG) binds with its cognate receptor (GCGR) and triggers the downstream pathway. This pathway follows two routes; in the first one, the suppressor of MEK2 (SMEK2) complex dephosphorylates cAMP-response element binding protein (CREB) regulated transcriptional coactivator 2 (CRTC2) that subsequently binds with CREB and induces the transcription of target genes. The second route follows the guanine protein subunit α Q (GNAQ) and involves the Forkhead box protein O1 (FoxO1) signaling pathway, where protein arginine N-methyltransferase 1 (PRMT1) methylates FOXO1, which forms a complex with peroxisome proliferator-activated receptor gamma coactivator 1-α (PGC-1α) and induces the gluconeogenesis-related genes. *B*, the workflow that was adopted to perform the LC-MS/MS study. CBP, CREB-binding protein; Crtc2^LKO^, liver-specific Crtc2 knockout; FASP, filter-aided sample preparation; PD, proteome discoverer; PP4C, protein phosphatase 4 catalytic subunit; SDS, sodium dodecyl sulfate.

TMT-based quantitative proteomics was used to profile changes in protein abundance and posttranslational modifications, such as phosphorylation and acetylation, in liver tissue extracts from HFD-fed mice. Comparative MS analysis of these extracts led to the identification of 5919 proteins from triplicate experiments (Fig. 1, Supplemental Fig. S1). Each identified protein contained at least two peptides matched to the proteome database, including at least one unique peptide. Among the identified proteins, 3246 were significantly expressed (Student’s *t* test *p*-value <0.05) and subsequently evaluated for differential abundance (log2FC >±0.26). The abundance levels remained unaffected by Crtc2^LKO^ for most of the identified proteins; however, 56 proteins were upregulated, and 59 proteins were downregulated. For phosphoproteome analysis, we identified 11,449 phosphopeptides from triplicate experiments (Supplemental Fig. S1). Among them, high-confidence phosphorylated sites, with a *p*-value <0.05 and site probability >75%, were used to enrich DPP candidates. Among these, 14 phosphorylated peptides were upregulated and 54 were downregulated. A comparison of the identified phosphorylation sites with those from PHOSIDA (25), PhosphoELM (26), and PhosphoSitePlus (27) indicated that 3537 (51.57%), 1550 (22.6%), and 471 (6.86%) sites, respectively, were previously unidentified. From acetylome analysis, 5604 acetylated peptides were identified in triplicate. Among these, 246 peptides were DAcPs, among which 69 were upregulated and 177 were downregulated (Supplemental Fig. S1). All DAPs, DPPs, and DAcPs were used for GO and KEGG biological pathway analyses.

**Fig. 2:**
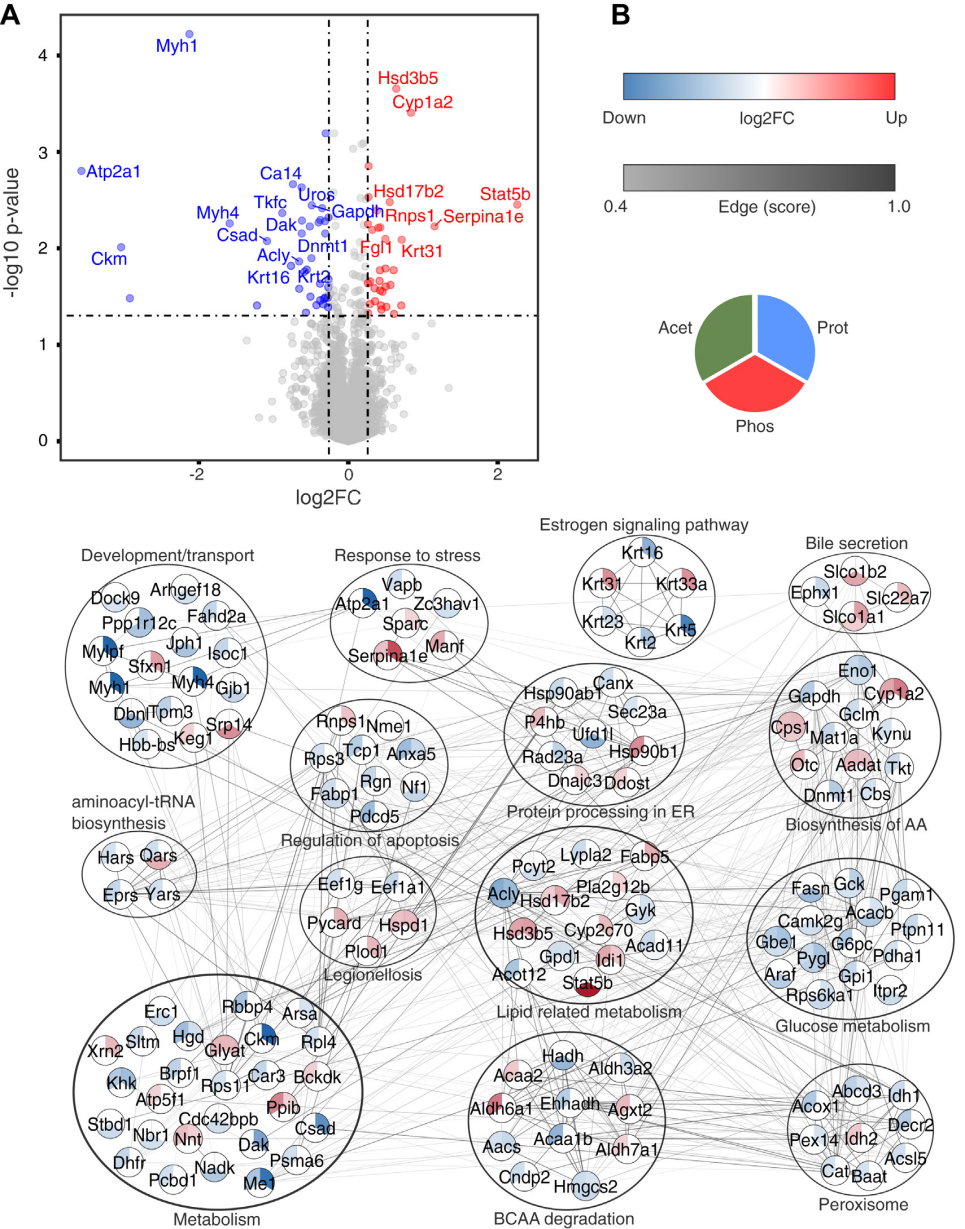
**Volcano plot and PPI network.** Differentially regulated proteins from independent datasets and enrichment analysis of Crtc2^LKO^. *A*, the volcano plot shows the log2 fold change (log2FC) *versus* the *p*-value plot (-log_10_*p*-value). *B*, protein–protein interaction network of differentially expressed (log2FC > ±0.26 with -log10pvalue >1.3), differentially phosphorylated (log2FC > ±0.26 with -log10pvalue >1.3), and differentially acetylated (log2FC > ±0.26 with −log10pvalue >1.3) proteins from Crtc2^LKO^ phenotype. The PPI network was created using the STRING app in Cytoscape with a 0.4 confidence level and without additional interactions. The edge color refers to the strength of interaction. The node-filled color corresponds to the log2FC value, where *blue* indicates downregulated, *red* indicates upregulated, and *white* is nonsignificant expression/nonidentification. The proteins are grouped based on mouse KEGG pathways, and single nodes are hidden for clarity. Acet, acetylome; Phos, phosphoproteome; Prot, proteome.

The volcano plot for the entire dataset and the PPI network for the DRPs (henceforth, DRPs refer to the aggregated list of DAPs, DPPs, and DAcPs) are shown in Figure 2. It is evident from the volcano plot (Fig. 2*A*) that the number of DRPs was less than that of the total proteins, and a higher number of proteins were downregulated across these datasets. The PPI of DRPs was created using STRINGv11.0 with a 0.4 confidence score and without additional interactions (Fig. 2*B*). Most proteins have mutual interactions, indicating their similar roles in cells. EnrichR was used for enrichment analysis; proteins were enriched in metabolic pathways (Table 1, Supplemental Tables S4 and S5, Supplemental Fig. S2) (16). The significantly enriched pathways, including valine, leucine, and isoleucine degradation pathways, are listed (Table 1). The degradation of BCAAs such as valine, leucine, and isoleucine is critical for many processes such as liver cirrhosis, renal failure, cancers, and diabetes (28). The molecular signature database and biological processes in gene ontology indicated that many hallmarks and pathways are metabolism-related (Supplemental Tables S4 and S5). The over-representation analysis consistently indicated that DRPs from the liver tissue of Crtc2^LKO^ are mainly involved in metabolism.

**Table 1:** Gene Ontology Enrichment Analysis of uniquely merged DRPs in Crtc2^LKO^ mice

Index	Name	*p*-value	Adjusted *p*-value	Odds ratio	Combined score
1	Valine, leucine, and isoleucine degradation	6.750e-11	2.045e-8	19.84	464.66
2	Pyruvate metabolism	4.243e-8	0.000002571	20.47	347.45
3	Tryptophan metabolism	1.022e-8	0.000001032	18.52	340.72
4	Peroxisome	2.425e-10	3.674e-8	14.55	322.14
5	Fatty acid degradation	1.432e-8	0.000001085	17.78	321.09
6	Glycolysis/Gluconeogenesis	1.528e-7	0.000006612	13.27	208.22
7	beta-Alanine metabolism	0.000009235	0.0003109	17.36	201.26
8	Fatty acid biosynthesis	0.0002426	0.004324	23.81	198.19
9	PPAR signaling pathway	7.253e-8	0.000003663	11.76	193.40
10	Butanoate metabolism	0.00009471	0.002208	16.46	152.50

The analysis was conducted using EnrichR, and pathways are arranged in decreasing order of combined score. The table contains the pathways from KEGG 2019 (mouse) with only the top 10 pathways provided and arranged in descending order of the combined score.

### Crtc2^LKO^ Disrupts the Glucagon and Insulin Signaling Pathways

To confirm the role of CRTC2 in metabolism, functional class scoring and SPIA were performed. The functional class scoring was calculated using the gene set enrichment analysis tool in R for all three proteomic datasets (Table 2). In the proteome and phosphoproteome, metabolism-related pathways, such as the glucagon signaling pathway, AMPK signaling pathway, and insulin signaling pathways, were enriched. For the acetylome dataset, in addition to the metabolism-related pathways, peroxisome and peroxisome PPAR signaling pathways are notable. Peroxisomes are small organelles that are critical in redox reactions and lipid homeostasis (29), whereas PPAR-mediated pathways play important roles in differentiation, development, metabolism, and tumorigenesis (30).

**Table 2:** Functional class scoring of DRPs from Crtc2^LKO^ phenotypes

CRTC2	Description (KEGG ID)	setSize	Enrichment score	NES	p.Adjust	Qvalues	Rank
Proteome	Glucagon signaling pathway (mmu04922)	26	−0.6898	−1.92296	0.027373	0.021457	371
	AMPK signaling pathway (mmu04152)	28	−0.63204	−1.7968	0.040102	0.031435	395
	Insulin signaling pathway (mmu04910)	33	−0.60364	−1.76805	0.040102	0.031435	371
Phospho-proteome	Glucagon signaling pathway (mmu04922)	26	−0.6898	−1.90507	0.036445	0.027752	371
	Insulin signaling pathway (mmu04910)	33	−0.60364	−1.75226	0.036445	0.027752	371
	AMPK signaling pathway (mmu04152)	28	−0.63204	−1.76959	0.036445	0.027752	395
	Spliceosome (mmu03040)	25	0.640773	2.007064	0.047851	0.036437	315
	Regulation of actin cytoskeleton (mmu04810)	33	−0.57894	−1.68056	0.047851	0.036437	119
Acetylome	Peroxisome (mmu04146)	41	−0.60487	−1.93962	0.004326	0.003624	150
	Glycolysis/Gluconeogenesis (mmu00010)	29	−0.64915	−1.95348	0.004326	0.003624	127
	PPAR signaling pathway (mmu03320)	23	−0.68808	−1.95795	0.004326	0.003624	201
	Biosynthesis of amino acids (mmu01230)	33	−0.56007	−1.72779	0.025081	0.021013	134
	Carbon metabolism (mmu01200)	68	−0.4784	−1.66409	0.025081	0.021013	134
	Biosynthesis of cofactors (mmu01240)	52	−0.49856	−1.66692	0.027597	0.023121	182

Functional class scoring was calculated by inputting all the proteins in the order of log2FC values using GSEA in R. This enrichment method does not depend on the DEPs only; therefore, it is useful when the number of DEPs is low. For this analysis, independent data was used, and the significant pathways are listed. All three data types generated similar significant pathways with metabolic pathways being dominant. Pathways with p.adjust < 0.05 are given. NES, normalized enrichment score.

Metabolic pathways are predominantly enriched in Crtc2^LKO^ mice; however, SPIA revealed that, based on the protein abundance trends, these pathways are inhibited (Table 3), particularly glucagon, insulin from the proteome-phosphoproteome dataset, and PPAR signaling for proteome-acetylome datasets (global probability of false discovery rate <0.05). Multiple proteomic approaches validated that Crtc2^LKO^ mice experience alterations in glucose and lipid metabolism under HFD, and these changes are perpetuated by the glucagon, insulin, and PPAR signaling pathways.

**Table 3:** Signaling pathway impact analysis

Name (KEGG ID)	pSize	NDE	pPERT	pG	pGFDR	Status
Proteome and phosphoproteome						
Glucagon signaling (mmu04922)	54	14	0.962	1.66E-06	0.00024	Inhibited
Insulin signaling (mmu04910)	78	14	0.32	5.00E-05	0.00358	Inhibited
Maturity onset diabetes of the young (mmu04950)	5	3	0.3287	0.002815	0.13419	Activated
Central carbon metabolism in cancer (mmu05230)	32	6	0.2733	0.007039	0.25163	Activated
Proteome and acetylome						
PPAR signaling (mmu03320)	47	14	1	5.29E-06	0.00067	Inhibited
Glucagon signaling (mmu04922)	54	10	0.3053	0.003223	0.20306	Activated
Central carbon metabolism in cancer (mmu05230)	32	7	0.6513	0.011679	0.3698	Activated

All three datasets were uniquely merged for the background list of genes. For the differentially expressed genes, the shared genes between proteomics and phosphoproteome with log_2_FC values >0.26 (or < −0.26) were used with nB = 3000, and 209 KEGG mouse pathways were searched. The pathways with global probability (pG) > 0.05 are given; however, the significant pathways are underlined.

### CRTC2 Interacts With SREBF1 to Modulate the Transcription of Glucose and Lipid Metabolism–Related Proteins

Six proteins, cytochrome P450 member 1A2 (CYP1A2; caffeine metabolism), kynurenine/α-aminoadipate aminotransferase (protein metabolism), NADPH-dependent 3-keto-steroid reductase Hsd3b5 (HSD3B5; testosterone metabolism), 1,4-α-glucan-branching enzyme (GBE1; required for normal glycogen accumulation), solute carrier organic anion transporter 1A1 (SLCO1A1; Na+-independent organic anions transporter), and ACLY (fatty acid metabolism), were identified in more than one dataset and were termed seed proteins. Among these, SLCO1A1 and ACLY were identified in proteome and phosphoproteome, while the CYP1A2, kynurenine/α-aminoadipate aminotransferase, HSD3B5, GBE1 and ACLY were identified in proteome and acetylome, with ACLY is the only protein that was identified in all the datasets. Almost all these proteins are involved in cellular metabolism in various forms except SLCO1A1, which highlights the pivotal role of CRTC2 in metabolism regulation. Moreover, this set of proteins offers an opportunity to narrow down the proteome to a limited number of targets to explore the regulatory mechanism in detail.

The seed proteins were used to create a new PPI network using STRING (25 additional interactions at 0.9 confidence level) and GeneMANIA (with auto-weighting and 25 additional interactors) and was called local-PPI (LPPI). In STRING, the LPPI consists of three subnetworks, and no interaction was identified for SLCO1A1 (Supplemental Fig. S3*A*); however, GeneMANIA integrated all proteins into a single network (Supplemental Fig. S3*B*). The newly identified proteins were queried against our datasets; only fatty acid synthase (FASN) was identified in both networks, as well as in our dataset (Table 4, Supplemental Table S6). In addition, the commonly identified proteins, CYP2D9 and CYP7B1, were removed from the subsequent analysis because they were not detected in any dataset.

**Table 4:** Identified proteins from LPPI using STRING and GeneMania

Type	STRING	GeneMania
Input protein	Slco1a1	Slco1a1
	Hsd3b5	Hsd3b5
	Acly	Acly
	Aadat	Aadat
	Gbe1	Gbe1
	Cyp1a2	Cyp1a2
Overlap with our data	Fasn	Fasn
	Pygl	G6PC
	Kynu	Hgd
	Acacb	Aass
	Ephx1	
	Pygb	
	Acaca	
	Cs	

The newly identified proteins are compared with our data and the overlapped proteins are given below. Among the newly identified proteins, FASN is the only protein that is common as well as identified by this study. The complete list of proteins is given in Supplemental data (Supplemental Table S6).

**Fig. 3:**
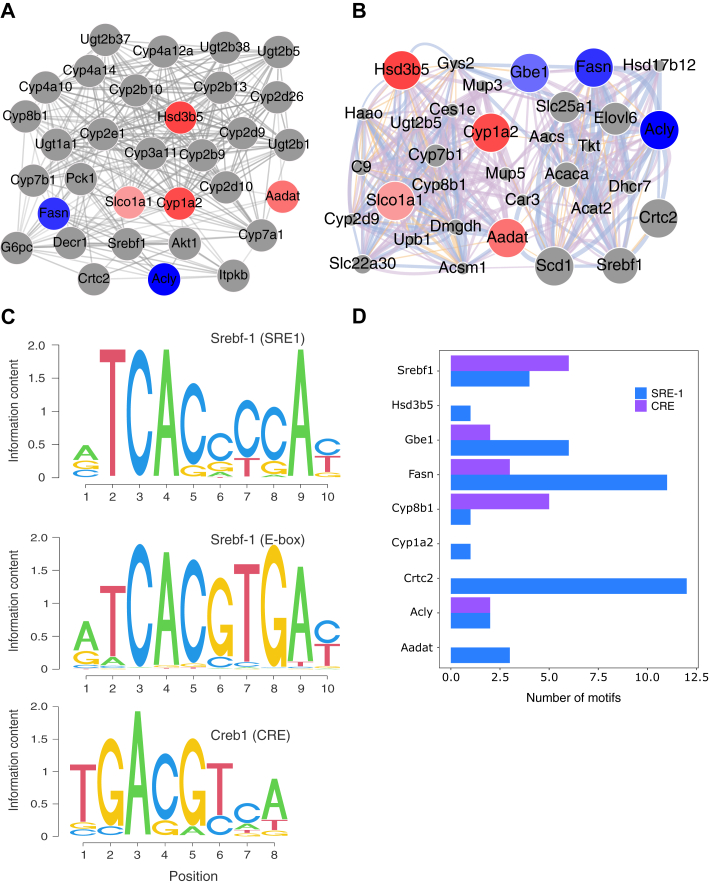
**CRTC2 may interact with SREBF1.** To study the possible mode of interaction between seed proteins, FASN, and CRTC2, these proteins were subjected to (*A*) STRING (0.40 confidence level and 25 additional interactors) and (*B*) GeneMania (for 25 additional interactors based on automatic weighting from different databases). Among the new interacting partners, SREBF1 is the only protein that has a direct interaction with CRTC2. The *red* nodes indicate upregulated, *blue* nodes indicate downregulated in most of the datasets, while the *gray* nodes indicate possible interacting proteins. GBE1 is hidden in the STRING network because it lacks any interaction. *C*, position weight matrix for motif identification in the promoter. SREBF1 can bind with two motifs; therefore, both have been searched. *D*, the number of motifs found in seed proteins, CRTC2 and SREBF1. The motif details regarding E-box are not given here due to lack of sufficient information. Further details can be found in Supplemental Table S8. CRTC, CREB-regulated transcriptional coactivator; GBE, glucan-branching enzyme; SREBF1, sterol regulatory element binding transcription factor 1.

To link CRTC2 with seed proteins, a third PPI (LPPI-CRTC2) was created, including FASN and CRTC2, using STRING and GeneMANIA (25 additional interactors at default confidence/weighting) (Fig. 3, *A* and *B*, Supplemental Table S7). In STRING, CRTC2 has five direct interaction partners: glucose-6-phosphatase catalytic subunit, phosphoenolpyruvate carboxykinase (PCK1), sterol regulatory element binding transcription factor 1 (SREBF1), AKT serine/threonine kinase 1, and inositol-trisphosphate 3-kinase B (Fig. 3*A*); however, in GeneMANIA, there were only two direct interaction partners (SREBF1 and ACLY) (Fig. 3*B*). SREBF1 was the common interacting partner in both networks (STRING score 2.775; GeneMANIA score (log_10_) −5.126). Both CRTC2-CREB (CRE) and SREBF1 (sterol regulatory element (SRE-1) and E-BOX1) are DNA-binding proteins; therefore, binding motifs were identified in the promoter regions (±5kb from the transcription start site) of genes from LPPI-CRTC2 (Fig. 3*C*). There were multiple genes with SRE-1–binding motif in their promoters; this included Fasn, Acly, Gbe1, Cyp2e1, and Crtc2 (Fig. 3*D*, Supplemental Tables S5 and S7); Acly and Cyp2e1 contained one and four E-box motifs, respectively. Crtc2 had the highest number of SRE-1 with no CRE motifs, suggesting its transcriptional regulation by SREBF1. Srebf1 had a higher number of CRE motifs indicating cross-activation. In addition, Fasn, Acly, and Srebf1 have motifs for both transcription factors, indicating potential dual regulation by these two factors. Based on data from the ENCODE database (31, 32), SREBF1 and CREB1 induced the expression of 1595 genes and 13,251 genes, respectively, where 1559 (97.75% and 11.76% with respect to SREBF1 and CREB1 target genome, respectively) genes from SREBF1 overlapped with CREB1. Of the overlapped genes, 286 (18.34%) genes were detected, and 33 (2.12%) genes were differentially regulated in Crtc2^LKO^ and most of the regulated genes were enriched in metabolism pathways (Supplemental Table S9).

Furthermore, the ChIP-Atlas webserver (https://chip-atlas.org) provides the relationship between transcription factors and their target genes using ChIP-seq data (33). Thus, to better understand the coregulation, the target gene lists from the ChIP-Atlas have been downloaded for CREB1 and SREBF1 (*M.musculus* [mm10] with region spanning ±10kb from the transcription start site). To remove the low confidence target genes, the data were filtered using average Model-based Analysis of ChIP-seq (MACS2) score (−10∗log10[MACS2 *Q*-value]) < 50 resulting in 11,854 and 2177 target genes for CREB1 and SREBF1, respectively. CREB1 and SREBF1 shared 1820 induced genes (15.35% with respect to CREB1 and 83.6% with respect to SREBF1); of those, 671 were detected in Crtc2^LKO^ dataset and 10 were differentially expressed with no significantly enriched pathway (p.adjusted value < 0.05). This further point out the plausibility that CRTC2 and SREBF1 can coregulate a subset of these genes (Supplemental Table S9).

CRTC2 may interact with SREBF1 similarly to CREB1. To analyze this, SREBF1 (PDB ID:1AM9 (34)) was superimposed onto CREB in the CRTC2–CREB–CRE complex (PDB ID:5ZKO (35)). The C-terminal regions of CREB (296–335 residues) and SREBF1 (358–397 residues) were superposed with a root mean square deviation of 0.96 Å (Cα atoms); however, the N-terminal deviated (Supplemental Fig. S4*A*). CREB has a long helical structure, whereas SREBF1 is divided into two helices connected by a loop, with the second part at an angle from the first. Twelve CREB residues interacted with CRTC2; among those, four (33.3%) were identical in SREBF1 (Y307, L325, K333, and L335 numbering is from CREB) (Supplemental Fig. S4*B*). Following the alignment, the similarity of the interacting residues was calculated using Sneath for comparison (36) (Supplemental Fig. S4*C*), where the most dissimilar residue pair was Asp-Trp with a value of 45. In addition, the bound DNA in each complex was poorly aligned and tilted at an angle of ∼35 − 40°. The transcription complex was large and contained multiple proteins; therefore, adjustment of relative and spatial orientation was expected. Based on structural and functional similarities, there is strong evidence of PPIs between CRTC2 and SREBF1 in transcriptional complex formation, where CRTC2 could modulate the transcriptional landscape of SREBF1 for a subset of genes in a certain metabolic environment. For instance, the altered gene expression of 33 genes (2.12%) is mostly from the proteomics dataset (16/33), indicating that loss of expression rather than alteration of posttranslational modification reinforces the transcriptional landscape modulation.

**Fig. 4:**
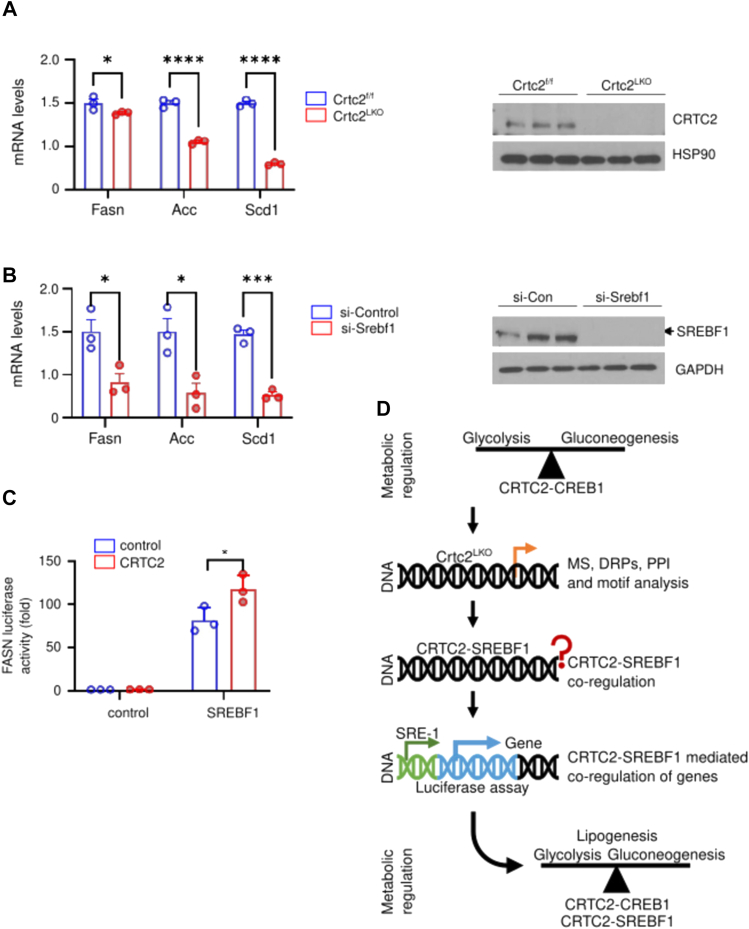
**CRTC2-SREBF1 coregulation is evident in luciferase assay.***A*, Q-PCR results showing expression of lipogenic genes (*Fas*, *Acc*, *Scd1*) in Crtc2^f/f^ or Crtc2^LKO^ hepatocytes (*B*), or in AML12 hepatocytes treated with si-Control or si-Srebf1. Relative expression of CRTC2 or SREBF1 was also shown by Western blot analysis (on the *right* of each panel). Data represent mean ± SEM (n = 3 biological replicates). *p* values were determined using unpaired student *t* test. ∗; *P*＜0.05, ∗∗∗; *P*＜0.001, ∗∗∗∗; *P*＜0.0001. *C*, luciferase assay was performed in HEK293T cells to determine the effect of CRTC2 and SREBF1 on FAS promoter (n = 3 biological replicates). Data represent mean ± SD (n = 3 biological replicates). *p* values were determined using unpaired student *t* test. ∗*P*＜0.05. *D*, summary figure. The key findings of the paper in a schematic manner. The CRTC2^LKO^ mice tissues were studied that prompted a hypothesis regarding coregulation of CRTC2-SREBF1 that was later verified through luciferase assay. CRTC, CREB-regulated transcriptional coactivator; SREBF1, sterol regulatory element binding transcription factor 1.

Finally, the proposed computational model has been validated using knocking out and luciferase assay in hepatocytes, where either Crtc2^LKO^ (Fig. 4*A*, left panel for mRNA expression, and right panel shows the protein expression using Western blot) or siRNA-mediated knockdown of Srebf1 significantly reduced the expression of lipogenic genes such as Fasn, acetyl-CoA carboxylase (Acc), and Stearoyl CoA desaturase 1 (Scd1) (Fig. 4*B*, left panel for mRNA expression,and right panel shows the protein expression using Western blot). Moreover, when a reported construct containing a natural SRE-1 motif (37) was used for the Fasn-luciferase assay, it showed that the luciferase activity significantly increased with the addition of CRTC2 indicating the coregulation of target genes (Fig. 4*C*). Here, the coregulation has been studied for a limited number of genes to prove the hypothesis; however, it should note that many other genes can also follow a similar mechanism that warrants further studies. A schematic representation of these steps has been shown in Figure 4*D* outlining the motives, methods, and outcome of the study.

### Crtc2^LKO^-Replicated Phenotypic Alterations in Animal Models

**Fig. 5:**
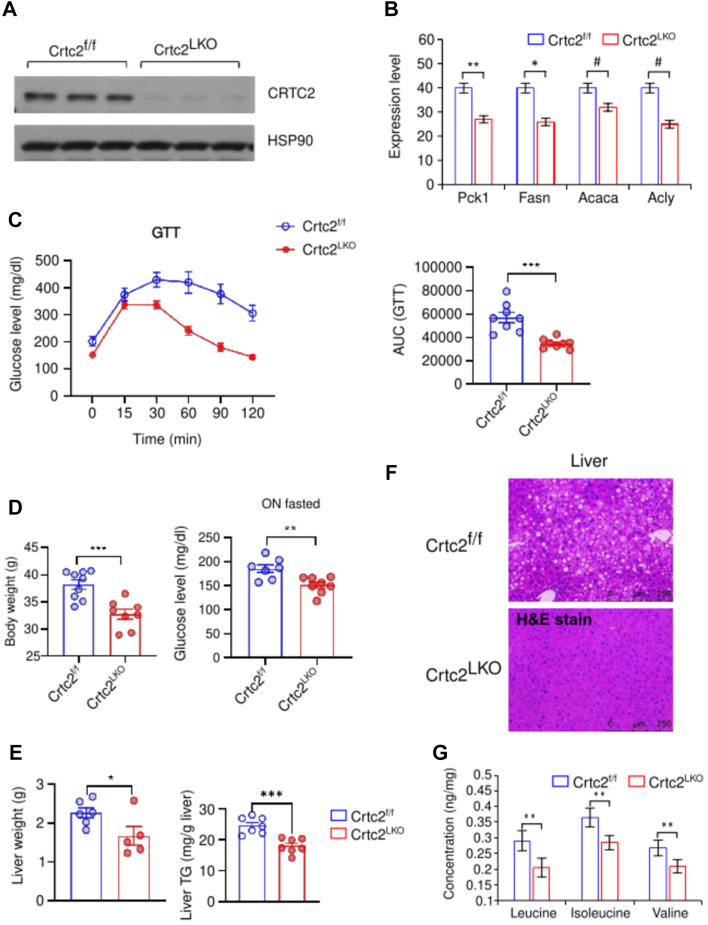
**Crtc2**^**LKO**^**disrupts glucose and fatty acid metabolism.** The effect of liver-specific depletion of Crtc2 on mice with 8-weeks high-fat diet feeding. *A*, the protein expression of Crtc2 was not detected in Crtc2^LKO^ when analyzed using Western blot, and HSP90 was used as an internal control. *B*, the RNA expression of metabolic proteins was analyzed; Crtc2^LKO^ significantly disrupted the Pck1, Fasn, Acaca, and Acly RNA expression. *C*, glucose tolerance test showing effects of Crtc2^LKO^ on glucose homeostasis. Data represent mean ± SEM (n = 8 mice per group). AUC was also shown. *D*, body weight (*left*) and 16-h fasting blood glucose levels (*right*) of Crtc2^f/f^ mice and Crtc2^LKO^ mice with 8-weeks high-fat diet feeding. Data on the *right* represent mean ± SEM (n = 9 for Crtc2^f/f^ mice and n = 8 for Crtc2^LKO^ mice), while the data on the *left* represent mean ± SEM (n = 7 for Crtc2^f/f^ mice and n = 8 for Crtc2^LKO^ mice). *E*, liver weight (*left*) and liver TG level (*right*) of Crtc2^f/f^ mice and Crtc2^LKO^ mice with 8-weeks high-fat diet feeding. Data on the *right* represent mean ± SEM (n = 6 for Crtc2^f/f^ mice and n = 5 for Crtc2^LKO^ mice), while the data on the *left* represent mean ± SEM (n = 7 for Crtc2^f/f^ mice and n = 7 for Crtc2^LKO^ mice). *F*, histological changes in the liver were examined through H&E staining. Representative data was shown. *G*, BCAAs were quantified as described in the Experimental Procedures section. These amino acids were significantly lower in Crtc2^LKO^. For each experiment, at least three independent observations were made. *p* values were determined using an unpaired student *t* test. ∗*P*＜0.05, ∗∗*P*＜0.01, ∗∗∗*P*＜0.001. ACACA, acetyl-CoA carboxylase α; AUC, area under curve; BCAA, branched-chain amino acid; CRTC, CREB-regulated transcriptional coactivator; GTT, glucose tolerance test; PCK1, phosphoenolpyruvate carboxykinase; TG, triglyceride.

The proteome data set indicated that Crtc2^LKO^ altered metabolic pathways; therefore, we validated this phenotype alteration and the impact of Crtc2^LKO^ in an animal model. Western blotting analysis indicated that CRTC2 was not expressed in the liver tissue of Crtc2^LKO^ compared with that in the control, confirming the successful knockout of CRTC2 in the liver (Fig. 5*A*). To verify whether changes at the protein level reflected that at the mRNA level, the mRNA levels of selected target genes of CRTC2 were analyzed. The mRNA levels of Pck1, Fasn, acetyl-CoA carboxylase α (Acaca), and ATP citrate synthase (Acly) were significantly downregulated in Crtc2^LKO^ mice (Fig. 5*B*). In the absence of CRTC2, these changes were expected and in line with proteomic data, indicating the downregulation of FASN (log2FC in prot/phospho/acetyl = ND/-0.61/NS), ACACA (log2FC = −0.35/−0.67/ND), and ACLY (log2FC = −0.658/−0.69/−0.81) (Fig. 5*B*). Although PCK1 was not detected in the LC-MS/MS data, it is an expected interactor of CRTC2 as shown in Figure 5*A*. The absence of PCK1 from the proteomics data might indicate the different cellular conditions when the cells were processed for proteomics data *versus* the cells were harvested for the RNA analysis. This further highlights the tight regulation of the metabolic environment.

These proteins, ACACA, ACLY, and FASN, are involved in *de novo* lipogenesis, and their reduction due to Crtc2 ablation causes a sharp drop in liver fat deposits and liver TG levels. For instance, Crtc2^LKO^ mice with HFD had significantly lower body weight and blood glucose levels in both glucose tolerance test (Fig. 5*C*, left; area under curve (AUC) in right panel) and fasting conditions (Fig. 5*D*), owing to the disruption of gluconeogenesis, compared to that in the control. Biological analysis reinforced that the liver-specific depletion of Crtc2 could significantly disturb glucose and lipid biosynthesis under HFD conditions, which is in line with earlier reports (11). The reduced level of these proteins also causes a sharp drop in liver weight (Fig. 5*E*, left panel), liver TG levels (Fig. 5*E*, right panel), and liver fat deposits (Fig. 5*F*). The level of BCAA was downregulated in the Crtc2^LKO^ model under HFD conditions (Fig. 5*G*); the reduction in glucose concentration triggered alternative energetic pathways such as BCAA degradation through kruppel-like factor 15 (KLF15) (Table 1, Supplemental Fig. S2) (38). The amount of KLF15 remained unaltered; however, multiple BCAA catabolic proteins including 3-Hydroxyisobutyrate dehydrogenase (HIBADH), aldehyde dehydrogenase 6a1 (ALDH6a1), alanine-glyoxylate aminotransferase 2 (AGXT2), branched-chain ketoacid dehydrogenase E1 (BCKDHs), isovaleryl-CoA dehydrogenase (IVD), and dihydrolipoamide branched-chain transacylase E2 (DBT) were upregulated. In contrast to this, dihydrolipoamide dehydrogenase (DLD), enoyl-coenzyme A, hydratase/3-hydroxyacyl Coenzyme A dehydrogenase (EHHADH), and hydroxyacyl-Coenzyme A dehydrogenase (HCDH) were downregulated (Supplemental Fig. S5).

## Discussion

Comprehensive proteome analysis was performed in Crtc2^LKO^ mice to study the impact of CRTC2 on energy homeostasis under metabolic stress (Fig. 1, *A* and *B*). The differentially abundant proteins were enriched in the metabolic pathways of glucose, proteins, and fatty acids (Tables 1 and 2). The results from the network topology-based method, SPIA, for common DRPs from proteome-phosphoproteome and proteome-acetylome reinforced the earlier observations and revealed that these pathways are inhibited (Table 3, Supplemental Tables S2 and S4), validating earlier reports (11, 39).

The elucidation of the pathway perturbation mechanism induced by the absence of CRTC2, using LPPI, further identified FASN as a mutual interactor (Supplemental Fig. S3, Table 4). FASN is a multifunctional enzyme involved in the biosynthesis and elongation of fatty acids, starting from a very basic unit (acyl-CoA). This unique property is critical for various cellular processes, and the importance of FASN in diverse pathologies is studied (40, 41). The identification of this protein shows that the isolated proteomic landscape accurately represents the phenotypic state of the cell. LPPI-CRTC2 was created to identify the possible interacting partners linking seed proteins commonly identified in three proteome datasets with CRTC2 under metabolic stress. SREBF1 has direct interaction with CRTC2 in both STRING and GeneMANIA PPIs (Fig. 3, *A* and *B*). SREBF1 is a key transcription factor that regulates the expression of genes that modulate cholesterol biosynthesis and lipid homeostasis (42).

The presence of binding motifs of CRTC2-CREB (CRE) and SREBF1 (SRE-1 and E-box motif) was identified in the promoter region of interacting proteins (Fig. 3*C*). Genes with a higher number of SRE-1 motifs included Crtc2, Fasn, Cyp2e1, and Gbe1, which are involved in glucose and lipid metabolism at various levels (Fig. 3*D*, Supplemental Table S8). The motif redundancy in their promoters indicates that their expression could be modulated by the SREBF1 transcriptional machinery. In addition, all three motifs were present only in Acly and Cyp2e1 (a member of the cytochrome P450 family of proteins). Both proteins are involved in fatty acid metabolism and play crucial roles in regulating fatty acid reservoirs in cells (43, 44, 45). Therefore, these genes require stringent regulation and could be transcribed under various cellular conditions to counter perturbations. The binding of SREBF1 with coactivators is known (46); from a structural point of view, SREBF1 can comfortably swap CREB when overlapped, because both CREB and SREBF1 interact with DNA through the basic leucine zipper (bZip) domain (Supplemental Fig. S4, *A*–*C*) (47, 48, 49). This computational model has been validated in experimental settings; for instance, when the knockout experiments were performed, it was evident that CRTC2 and SREBF1 influence the expression of lipogenic genes such as Fasn, Acc, and Scd1 (Fig. 4, *A* and *B*). Similarly, the luciferase activity was significantly increased when Crtc2 was added (Fig. 4*C*), indicating a cooperative regulation of critical genes. As the glucose and lipid metabolisms are linked at various points, it is rational that these proteins cooperate and tightly regulate the flow and appendages of the metabolic network at multiple levels.

Coactivator binding is transcription factor-dependent; the CREB-binding domain (CBD) of CRTC2 does not bind to DNA independently; however, it strongly binds to DNA in the presence of CREB (50). However, a crystallographic study showed that CBD binds to DNA, although weakly; in addition, it confers geometric preference (35). Therefore, the weak binding of CBD to SREBF1 is sufficient for the expression of a subset of target genes. Gene transcription is a combinatorial process that requires many proteins to produce the desired outcome (51, 52). The relative contribution of individual factors can drastically change the transcription patterns of various genes, which can be used to modulate cellular responses. A single factor can contribute to diverse functional responses at different levels of cellular genetic processes (53). Both CRTC2-CREB and SREBF1 induce the expression of lipid- and carbohydrate-related genes; therefore, CRTC2 could fine-tune relative activity and balance homeostasis, especially under metabolic stress (54). CRTC2 is active during fasting, while SREBF1 is highly induced under feeding conditions. Therefore, HFD-induced insulin resistance activates both proteins in the liver, supporting our hypothesis that these two factors function cooperatively to regulate the transcription of a subset of genes involved in lipid metabolism under metabolic stress. PPARγ coactivator-1β, a coactivator of PPARα in the regulation of fatty acid beta-oxidation during fasting conditions, could act as a coactivator of SREBF1 to regulate hepatic lipogenesis upon HFD feeding in mice (12) showing that this phenomenon is not unprecedented.

CRTC2-CREB may not bind to all CRE sites because the DNA electrostatic potential may confer selectivity (35). The interaction between CRTC2 and SREBF1 may not be universal as in the case of CRTC2–CREB complex, and for critical genes such as Acly and Fasn, various mechanisms could regulate their expression. For instance, both Acly and Fasn are also induced by SREBF1, and in the case of Crtc2^LKO^, transcription by either factor is hampered, resulting in downregulation. This dual regulation may be an evolutionary measure to ensure the conducive levels of glucose and lipids in cells (55). In addition to transcriptional regulation, phosphorylated CRTC2 is sequestered in the cytoplasm and is unable to interact with COPII-mediated SREBF1 maturation and transport (12). When the cell receives the signal, dephosphorylated CRTC2 performs dual functions: it forms a complex with CREB to induce gene expression and inhibits the maturation of SREBF1, which restricts cholesterol biosynthesis. Such differential functioning represents a finely regulated switch in metabolic pathways that ultimately regulates the concentration of various molecules in cells (54, 56).

CRTC2 is a co-activator that plays a vital role in glucose homeostasis. Therefore, in the absence of a coactivator, glucose metabolism–related genes were downregulated (Fig. 4*B*), which culminated in the disruption of glucose tolerance, body weight, lipid storage, liver weight, and TG levels (Fig. 5*C*) (8). Glucose is the primary energy source; therefore, glucose depletion triggers alternative energy sources such as BCAA. BCAA catabolism initiates energy homeostasis to compensate for metabolic perturbations (38) (Fig. 5*D*, Table 1). However, the depletion of BCAAs can accelerate the onset of liver cirrhosis, renal failure, cancer, and diabetes (57); whereas blocking BCAA catabolism can improve diabetic conditions (58). Activation of BCAA catabolism can induce adaptive thermogenesis in response to cold shock in brown adipose tissues, where SLC25A44 actively transports BCAAs into mitochondria, improving diet-induced obesity and glucose intolerance (59). Therefore, the regulation of BCAA-related proteins by CRTC2 is an attractive target for the regulation of various metabolic abnormalities (Fig. 5).

In summary, liver-specific knockout of Crtc2 in mice revealed that it is vital in glucose, lipid, and protein metabolism and may influence BCAA metabolism indirectly under metabolic stress in the liver. These diverse functions are executed by interacting with more than one transcription factor such as CREB and SREBF1, and the relative interplay between the induction of target genes can fine-tune cellular homeostasis under various metabolic conditions.

## Data Availability

The mass spectrometry proteomics data have been deposited to the ProteomeXchange Consortium *via* the PRIDE (60) partner repository with the dataset identifier PXD039817 and 10.6019/PXD039817.

## Supplemental data

This article contains supplemental data.

## Conflict of interest

The authors declare no competing interests.
