# Supplementary material for: CREB-Regulated Transcriptional Coactivator 2 Proteome Landscape is Modulated by SREBF1
Source: Mol Cell Proteomics. 2023 Aug 28;22(10):100637. doi: 10.1016/j.mcpro.2023.100637 (PMC10522995; doi:10.1016/j.mcpro.2023.100637)
Supplement: Supplemental Figures S1–S5 and Tables S1–S10 [file mmc1.docx]

Table of contents

**Title page**

**Supplemental Figure S1:** Global proteomic analysis

**Supplemental Figure S2**: Differentially expressed proteins from independent dataset and enrichment analysis of Crtc2^LKO^.

**Supplemental Figure S3:** Branched chain amino acid (BCAA) pathway.

**Supplemental Figure S4:** Local PPI (LPPI) network of seed proteins.

**Supplemental Figure S5:** Structural similarity between CRTC2-CREB and CRTC2-SREBF1 crystal structures

**Supplemental Table S1:** Global proteome data: Protein identification, accession, coverage, and quantification related data

**Supplemental Table S2:** Phosphopeptides: detailed information regarding peptide sequence, mass spectra, charges, site modification and probability and quality scores

**Supplemental Table S3:** Phosphopeptides: detailed information regarding peptide sequence, mass spectra, charges, site modification and probability and quality scores

**Supplemental Table S4:** Gene Ontology Enrichment Analysis of uniquely merged DEPs in Crtc2^LKO^ mice using MSigDB Hallmark (2020) dataset.

**Supplemental Table S5:** Gene Ontology Enrichment Analysis of uniquely merged DEPs in Crtc2^LKO^ mice using GO Biological Process (2018) dataset.

**Supplemental Table S6:** Protein from the seed network of Crtc2^LKO^ phenotype.

**Supplemental Table S7:** List of proteins and their scores from LPPI-CRTC2 network.

**Supplemental Table S8:** Identification of motif in promoter region.

**Supplemental Table S9:** Gene ontology (biological process) analysis of genes that were DEPs and the target of CREB and SREBF1 from ENCODE and ChIP-Atlas databases.

**Supplemental Table S10:** List of transcripts with at least one motif identified.

**CREB regulated transcriptional coactivator 2 proteome landscape is modulated by SREBF1**

**Authors:**

Jae Min Lim^1,*^, Muhammad Ayaz Anwar^1,*^, Hye-Sook Han^3^, Seung-Hoi Koo^3,#^, Kwang Pyo Kim^1,2,#^

**Affiliations:**

^1^Department of Applied Chemistry, Institute of Natural Science, Global Center for Pharmaceutical Ingredient Materials, Kyung Hee University, Yongin, 17104, South Korea

^2^Department of Biomedical Science and Technology, Kyung Hee Medical Science Research Institute, Kyung Hee University, Seoul, 02453 South Korea

^3^Division of Life Sciences, Korea University, 145 Anam-Ro, Seongbuk-Gu, Seoul 02841, South Korea

^#^**Correspondence:**

Prof. Kwang Pyo Kim

Department of Applied Chemistry, College of Natural Sciences, Kyung Hee University, Yong-in 446-701, South Korea. Email: [kimkp@khu.ac.kr](mailto:kimkp@khu.ac.kr)

Prof. Seung-Hoi Koo

Division of Life Sciences, Korea University, 145 Anam-Ro, Seongbuk-Gu, Seoul 02841, South Korea, Email:  [koohoi@korea.ac.kr](mailto:kimkp@khu.ac.kr)

**^*^Equally contributed**

**Running Title:** CRTC2 and SREBF1 in metabolic regulation

**Supplemental Figure S1:** **Global proteomic analysis.** The total number of proteins identified (left panel), and the coverage among the three replicates as in the form of Venn diagram (middle panel). On the right, the distribution of DEPs is given.


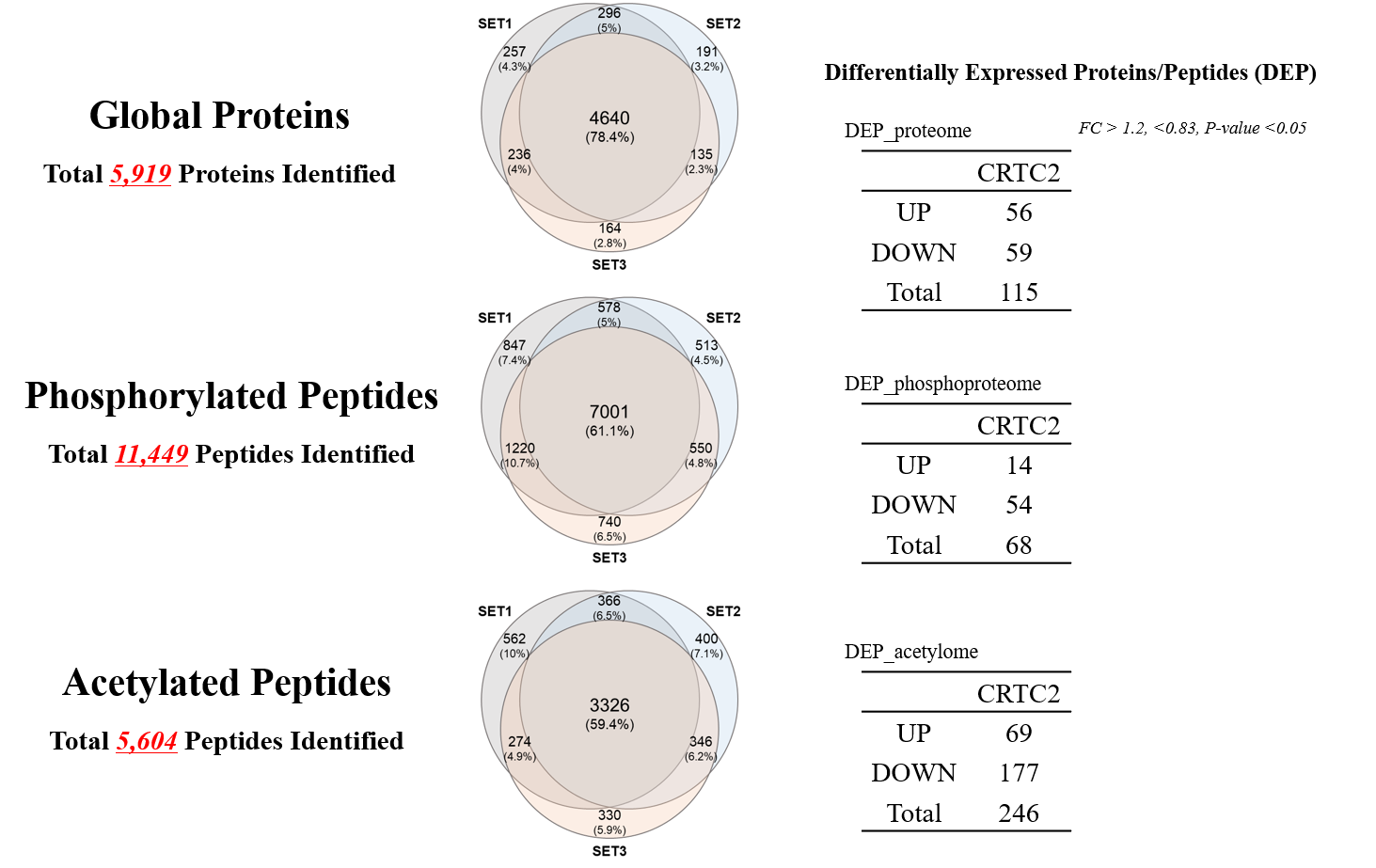


**Supplemental Figure S2**: **Differentially expressed proteins from independent dataset and enrichment analysis of Crtc2^LKO^**. The enrichment of biological process as calculated by EnrichR. The first twenty enriched KEGG pathway (KEGG 2019 mouse) have been given for each case. *Values with more than 1000 are capped.


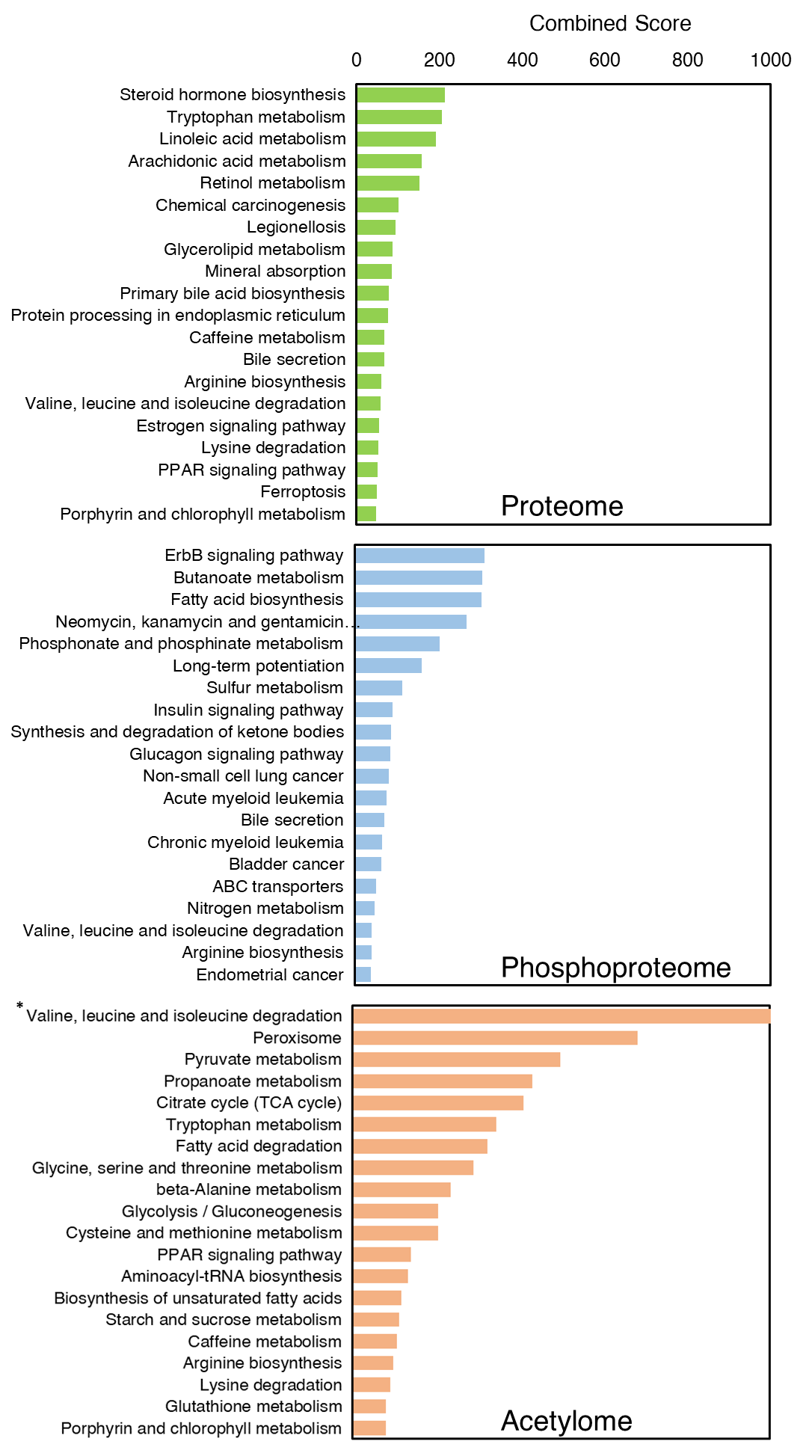


**Supplemental Figure S3: Local PPI (LPPI) network of seed proteins.** (A) The common genes between proteomics and phosphoproteomics data and between proteomics and acetylomics data have been isolated and LPPI network has been created using STRING with 25 additional interactions and a confidence level of 0.90 to extract only the highly confident interaction. The interacting attributes include text mining, co-expression, databases, experiment, co-occurrences, neighborhood, and fusion. (B) For GeneMania, the gene list was subjected to find out the other up to 25 related genes with equivalent number of annotations using default weighting matrix (automatic). Among the output network, the coexpression (pink edge color) and colocalization (blue) attributes account for 90.87% and 9.13% respectively. The red nodes are upregulated, blue nodes are downregulated in most of the datasets, while the grey are possible interacting proteins. The single nodes are hidden for clarity.


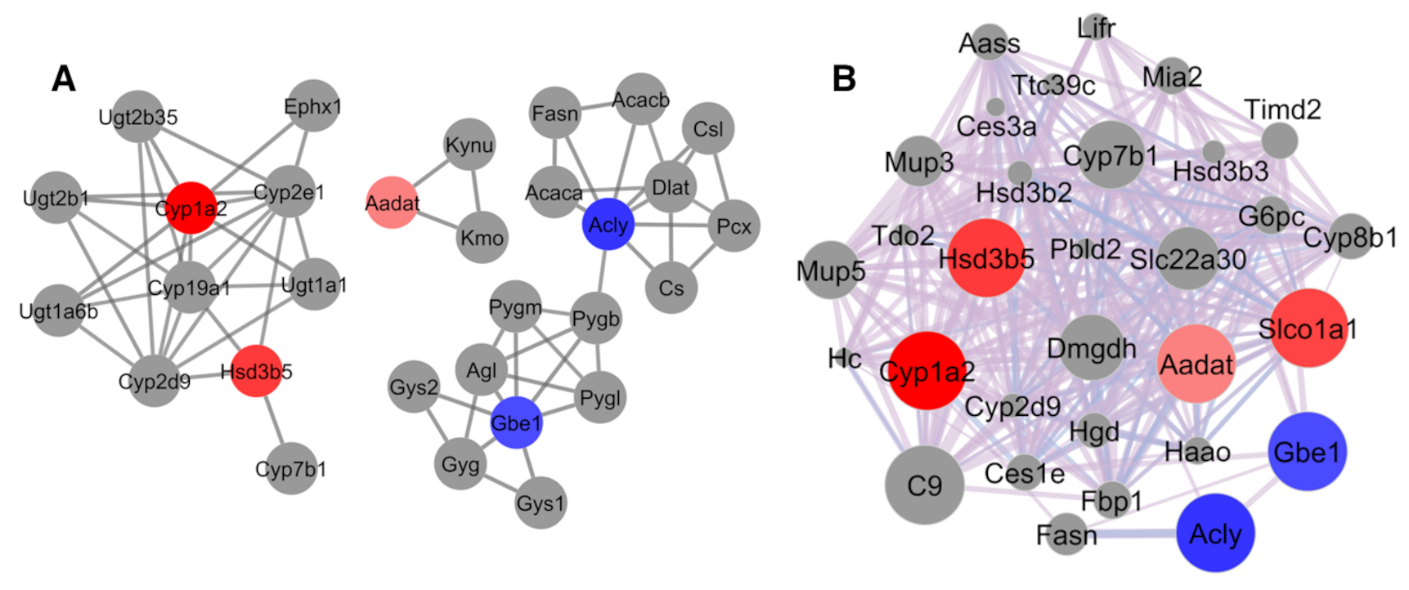


**Supplemental Figure S4: Structural similarity between CRTC2-CREB and Crtc2-Srebf1 crystal structures.** (**A**) The crystal structures from CRTC2-CREB-CRE complex (PDB ID: 5ZKO) and SREBF1-DNA complex (PDB ID: 1AM9 chain C, D) have been superposed to analyze the structural similarities. The C-terminal regions of CREB (296-335 residues) and SREBF1 (358-397 residues) were superposed with 0.96 Å RMSD. The superposition has been created using Chimera with best aligning chains using Needleman-Wunch alignment algorithm and BLOSUM-62 matrix. Secondary structure score was also included (30%) and matching was iterated until no pair exceeds 2.0 Å. DNA has been hidden for clarity. (**B**) The interacting residues from CREB that interact with CRTC2 are labelled and shown in purple stick while the residues from SREBF1 are shown in blue stick conformation. The numbering of residues follows CREB protein. (**C**) The residues from both CREB and SREBF1 are given with Sneath’s index of dissimilarity. The residues in green are identical in both proteins.


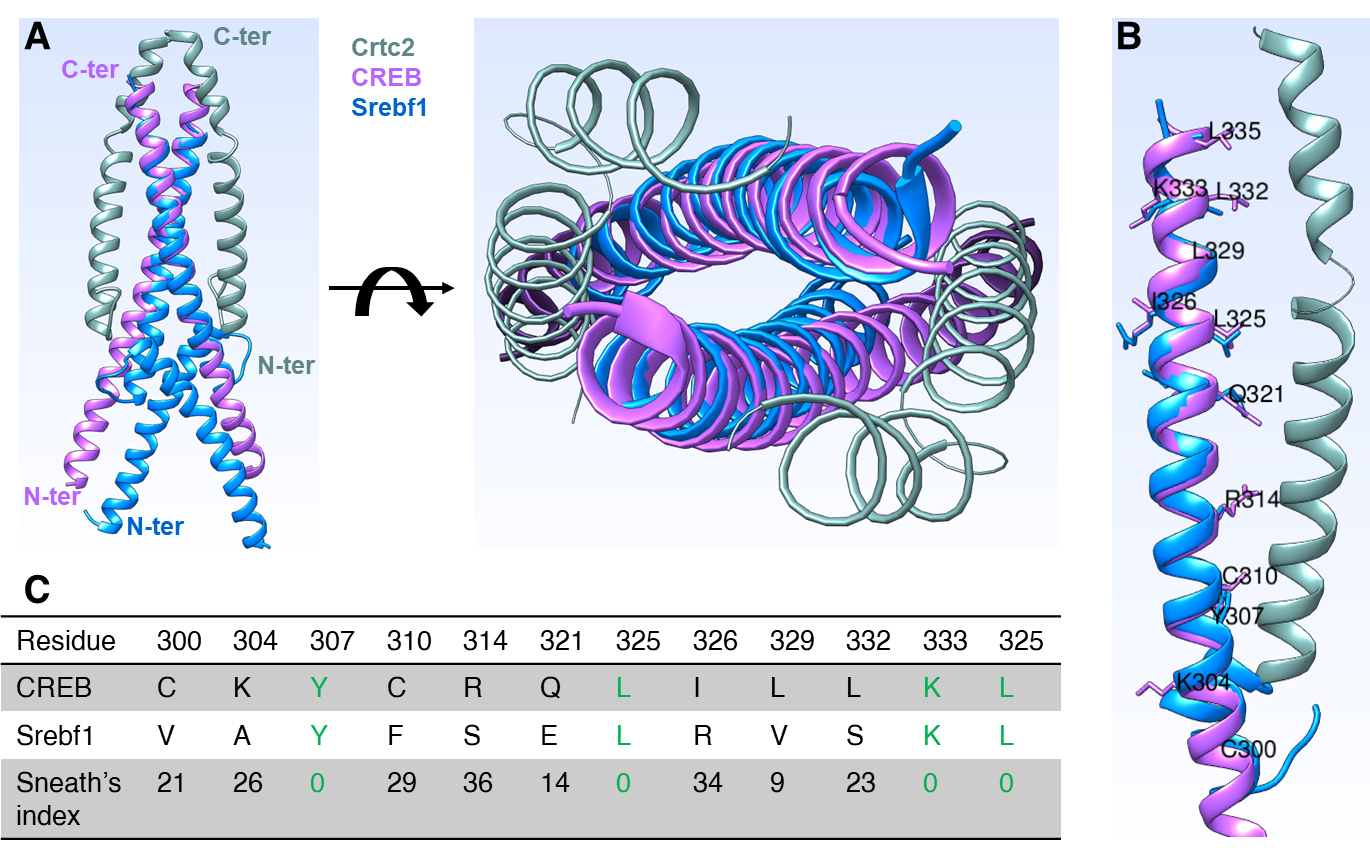


**Supplemental Figure S5: Branched chain amino acid (BCAA) pathway.** An overlay has been created using PathView from KEGG dataset to show the up/down regulated proteins in BCAA pathway.


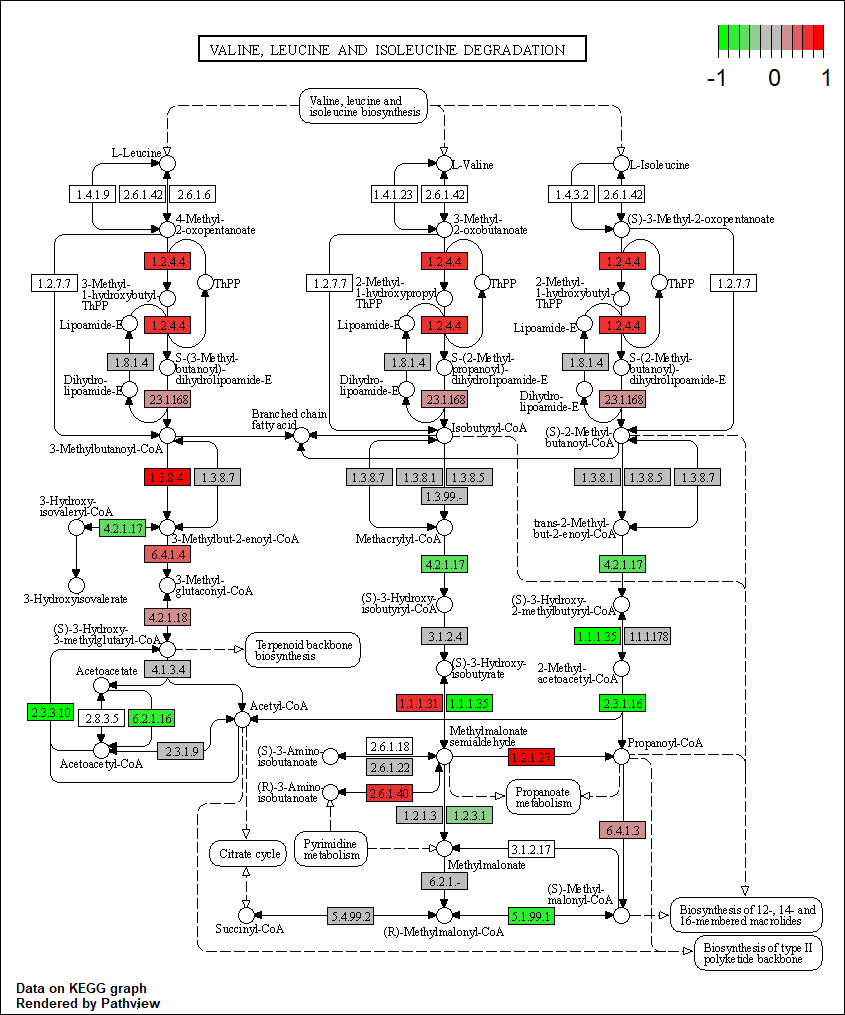


**Supplemental Table S4: Gene Ontology Enrichment Analysis of uniquely merged DEPs in Crtc2^LKO^ mice using MSigDB Hallmark (2020) dataset.** The analysis has been conducted using EnrichR and pathways are arranged with decreasing order of combined score.

| Index | Name | P-value | Adjusted p-value | Odds Ratio | Combined score |
| --- | --- | --- | --- | --- | --- |
| 1 | Fatty Acid Metabolism | 4.577e-15 | 2.289e-13 | 12.66 | 417.95 |
| 2 | Peroxisome | 3.411e-8 | 8.529e-7 | 10.68 | 183.69 |
| 3 | mTORC1 Signaling | 2.752e-7 | 0.000004587 | 6.67 | 100.70 |
| 4 | Xenobiotic Metabolism | 2.752e-7 | 0.000003440 | 6.67 | 100.70 |
| 5 | Bile Acid Metabolism | 0.000007896 | 0.00006580 | 7.94 | 93.25 |
| 6 | Cholesterol Homeostasis | 0.00005454 | 0.0003896 | 9.01 | 88.44 |
| 7 | Adipogenesis | 0.000002057 | 0.00002057 | 6.11 | 80.02 |
| 8 | Myogenesis | 0.0004686 | 0.002928 | 4.44 | 34.07 |
| 9 | Myc Targets V1 | 0.0004686 | 0.002603 | 4.44 | 34.07 |
| 10 | Glycolysis | 0.0004686 | 0.002343 | 4.44 | 34.07 |

**Supplemental Table S5: Gene Ontology Enrichment Analysis of uniquely merged DEPs in Crtc2^LKO^ mice using GO Biological Process (2018) dataset.** The analysis has been conducted using EnrichR and pathways are arranged with decreasing order of combined score.

| **Index** | **Name** | **P-value** | **Adjusted p-value** | **Odds Ratio** | **Combined score** |
| --- | --- | --- | --- | --- | --- |
| 1 | Tricarboxylic acid metabolic process (GO:0072350) | 0.000004258 | 0.001552 | 34.19 | 422.79 |
| 2 | Acetyl-CoA metabolic process (GO:0006084) | 0.000005919 | 0.002014 | 31.75 | 382.14 |
| 3 | Alpha-amino acid metabolic process (GO:1901605) | 2.986e-7 | 0.0005078 | 21.51 | 323.10 |
| 4 | 2-oxoglutarate metabolic process (GO:0006103) | 0.00001378 | 0.004395 | 26.14 | 292.61 |
| 5 | Glycolytic process through glucose-6-phosphate (GO:0061620) | 0.000002564 | 0.001189 | 22.22 | 286.09 |
| 6 | Canonical glycolysis (GO:0061621) | 0.000002564 | 0.001090 | 22.22 | 286.09 |
| 7 | Glucose catabolic process to pyruvate (GO:0061718) | 0.000002564 | 0.001006 | 22.22 | 286.09 |
| 8 | Glutathione biosynthetic process (GO:0006750) | 0.0001122 | 0.02044 | 30.30 | 275.62 |
| 9 | Monocarboxylic acid metabolic process (GO:0032787) | 2.571e-10 | 0.000001312 | 12.35 | 272.61 |
| 10 | Dicarboxylic acid metabolic process (GO:0043648) | 5.521e-8 | 0.0001409 | 15.07 | 251.78 |

**Supplemental Table S6:** **Protein from the seed network of Crtc2^LKO^ phenotype.** Seed genes are bold, while the common identified genes by each tool have been underlined. From each tool, the identified genes have been compared to the DE genes in our dataset, and the matched genes are red. In general, STRING identified a greater number of genes (i.e., 7) than the GeneMania (3). CYP2D9 and CYP7B1 were consistently identified by STRING and GeneMania, however, these were not identified in our datasets, thus has been excluded from subsequent analysis.

| **STRING** | **GeneMANIA** |
| --- | --- |
| **Slco1a1** | **Slco1a1** |
| **Hsd3b5** | **Hsd3b5** |
| **Acly** | **Acly** |
| **Aadat** | **Aadat** |
| **Gbe1** | **Gbe1** |
| **Cyp1a2** | **Cyp1a2** |
| Pygb | Slc22a30 |
| Cyp2d9 | Cyp7b1 |
| Cyp19a1 | Mup5 |
| Cyp2e1 | Mup3 |
| Gys1 | C9 |
| Gys2 | Cyp8b1 |
| Gyg | **Fasn** |
| Agl | G6pc |
| **Fasn** | Mia2 |
| Pygl | Dmgdh |
| Pygm | Aass |
| Kmo | Timd2 |
| Csl | Ces1e |
| Acaca | Hgd |
| Cs | Fbp1 |
| Kynu | Haao |
| Cyp7b1 | Cyp2d9 |
| Ugt2b1 | Hsd3b3 |
| Acacb | Tdo2 |
| Ugt1a1 | Ttc39c |
| Pcx | Pbld2 |
| Ephx1 | Hc |
| Ugt1a6b | Hsd3b2 |
| Ugt2b35 | Ces3a |
| Dlat | Lifr |

**Supplemental Table S7: List of proteins and their scores from LPPI-CRTC2 network.** The query proteins are bold. SREBF1 has substantially higher scores in both networks.

| **STRING** | | **GeneMANIA** | | |
| --- | --- | --- | --- | --- |
| **Gene name** | **Score** | **Gene name** | **Log Score** | **Type** |
| **Cyp1a2** | query | **Slco1a1** | -0.27095 | query |
| **Hsd3b5** | query | **Crtc2** | -0.27433 | query |
| **Slco1a1** | query | **Acly** | -0.53656 | query |
| **Fasn** | query | **Aadat** | -0.57032 | query |
| **Acly** | query | **Hsd3b5** | -0.57582 | query |
| **Crtc2** | query | **Gbe1** | -0.62696 | query |
| **Aadat** | query | **Cyp1a2** | -0.65538 | query |
| **Gbe1** | query | **Fasn** | -0.65769 | query |
| Cyp7a1 | 3.14 | Scd1 | -5.08326 | result |
| Cyp3a11 | 2.801 | Srebf1 | -5.12648 | result |
| Srebf1 | 2.775 | Elovl6 | -5.18643 | result |
| Akt1 | 2.701 | Slc25a1 | -5.2131 | result |
| Cyp2e1 | 2.683 | Acaca | -5.32315 | result |
| Cyp4a14 | 2.559 | Cyp7b1 | -5.37002 | result |
| Cyp2d9 | 2.526 | Slc22a30 | -5.44586 | result |
| Cyp4a10 | 2.456 | Dmgdh | -5.53048 | result |
| Decr1 | 2.383 | C9 | -5.53088 | result |
| Cyp4a12a | 2.373 | Dhcr7 | -5.56032 | result |
| Pck1 | 2.371 | Mup5 | -5.5753 | result |
| G6pc | 2.34 | Ces1e | -5.58443 | result |
| Cyp2b10 | 2.333 | Car3 | -5.60354 | result |
| Cyp8b1 | 2.329 | Acsm1 | -5.60622 | result |
| Cyp7b1 | 2.326 | Cyp2d9 | -5.60915 | result |
| Ugt2b1 | 2.319 | Mup3 | -5.61097 | result |
| Ugt2b38 | 2.31 | Haao | -5.61328 | result |
| Cyp2b13 | 2.29 | Aacs | -5.61423 | result |
| Cyp2b9 | 2.263 | Hsd17b12 | -5.61609 | result |
| Ugt1a1 | 2.245 | Tkt | -5.62346 | result |
| Cyp2d10 | 2.158 | Gys2 | -5.67515 | result |
| Ugt2b37 | 2.138 | Acat2 | -5.67719 | result |
| Ugt2b5 | 2.132 | Upb1 | -5.68421 | result |
| Cyp2d26 | 2.13 | Cyp8b1 | -5.70001 | result |
| Itpkb | 2.077 | Ugt2b5 | -5.70369 | Result |

**Supplemental Table S8: Identification of motif in promoter region.** These motifs have been identified with 90% or above confidence level using mouse genomic data. For the complete list of transcripts, see Supplemental Table S7. CRE, cAMP response element; Srebf1, sterol regulatory binding protein 1; SRE1, sterol regulatory element 1.

| **Gene** | **Srebf1 (E-BOX)** | **Srebf1 (SRE1)** | **CRE** |
| --- | --- | --- | --- |
| *Cyp1a2* | 0 | 1 | 0 |
| *Hsd3b5* | 0 | 1 | 0 |
| *Fasn* | 0 | 11 | 2 |
| *Acly* | 1 | 2 | 2 |
| *Crtc2* | 0 | 12 | 0 |
| *Aadat* | 0 | 3 | 0 |
| *Gbe1* | 0 | 6 | 2 |
| *Cyp7a1* | 0 | 2 | 0 |
| *Srebf1* | 0 | 4 | 6 |
| *Akt1* | 0 | 0 | 4 |
| *Cyp2e1* | 4 | 4 | 4 |
| *Cyp4a14* | 0 | 0 | 4 |
| *Cyp2d9* | 0 | 0 | 8 |
| *Cyp4a10* | 0 | 2 | 0 |
| *Cyp4a12a* | 0 | 2 | 1 |
| *G6PC* | 0 | 2 | 1 |
| *Cyp8b1* | 0 | 1 | 5 |
| *Ugt2b1* | 0 | 1 | 3 |
| *Ugt2b38* | 0 | 0 | 1 |
| *Cyp2b9* | 0 | 1 | 0 |
| *Itpkb* | 0 | 1 | 1 |

**Supplemental Table S9:** Gene ontology (biological process) analysis of genes that were DEPs and the target of CREB and SREBF1 from ENCODE and ChIP-Atlas databases.

| **ENCODE data** | | | |
| --- | --- | --- | --- |
| **Term** | **Overlap** | **Adjusted P-value** | **Genes** |
| secondary alcohol biosynthetic process (GO:1902653) | 4/34 | 5.11E-5 | IDI1; ACLY; CYP51A1; TM7SF2 |
| cholesterol biosynthetic process (GO:0006695) | 4/35 | 5.11E-5 | IDI1; ACLY; CYP51A1; TM7SF2 |
| sterol biosynthetic process (GO:0016126) | 4/38 | 5.11E-5 | IDI1; ACLY; CYP51A1; TM7SF2 |
| regulation of primary metabolic process (GO:0080090) | 5/130 | 1.94E-4 | IDI1; PSMA6; FASN; CYP51A1; TM7SF2 |
| cholesterol metabolic process (GO:0008203) | 4/77 | 5.37E-4 | IDI1; ACLY; CYP51A1; TM7SF2 |
| regulation of lipid metabolic process (GO:0019216) | 4/92 | 9.08E-4 | IDI1; FASN; CYP51A1; TM7SF2 |
| regulation of vascular associated smooth muscle cell apoptotic process (GO:1905459) | 2/7 | 0.002773 | DNMT1; SOD2 |
| tricarboxylic acid metabolic process (GO:0072350) | 2/12 | 0.007588 | ACLY; IDH1 |
| lipid biosynthetic process (GO:0008610) | 3/80 | 0.01206 | ACLY; PCYT2; CYP51A1 |
| acyl-CoA biosynthetic process (GO:0071616) | 2/17 | 0.01244 | ACLY; FASN |
| fatty-acyl-CoA metabolic process (GO:0035337) | 2/22 | 0.01911 | ACLY; FASN |
| fatty-acyl-CoA biosynthetic process (GO:0046949) | 2/30 | 0.03272 | ACLY; FASN |
| **ChIP-Atlas data** | | | |
| **Term** | **Overlap** | **P-value** | **Genes** |
| negative regulation of smooth muscle cell apoptotic process (go:0034392) | 1/6 | 0.002997 | DNMT1 |
| macromolecule depalmitoylation (go:0098734) | 1/7 | 0.003495 | LYPLA2 |
| protein depalmitoylation (go:0002084) | 1/7 | 0.003495 | LYPLA2 |
| growth hormone receptor signaling pathway via jak-stat (go:0060397) | 1/7 | 0.003495 | STAT5B |
| taurine metabolic process (go:0019530) | 1/7 | 0.003495 | STAT5B |
| microtubule anchoring (go:0034453) | 1/9 | 0.004492 | PEX14 |
| lipoprotein catabolic process (go:0042159) | 1/10 | 0.00499 | LYPLA2 |
| protein import into peroxisome matrix (go:0016558) | 1/13 | 0.006482 | PEX14 |
| regulation of multicellular organism growth (go:0040014) | 1/15 | 0.007476 | STAT5B |
| protein targeting to peroxisome (go:0006625) | 1/16 | 0.007973 | PEX14 |
